# Antibiotic-resistant pathogens and heavy metal-tolerant bacteria in *Batissa violacea*: A study from the Ba River, Fiji

**DOI:** 10.1371/journal.pone.0359203

**Published:** 2026-09-25

**Authors:** Divya Darshika Mala, Ashneel Ajay Singh, Ramesh Subramani, Sathiyaraj Srinivasan, Taitusi Taufa, Ashok Kumar, Francis Mani

**Affiliations:** 1 Discipline of Biological and Chemical Sciences, School of Agriculture, Geography, Environment, Ocean and Natural Sciences, The University of the South Pacific, Suva, Fiji; 2 Department of Biology and Environmental Technology, College of Natural Science, Seoul Women’s University, Seoul, Korea; PSTU: Patuakhali Science and Technology University, BANGLADESH

## Abstract

The Ba River in Fiji is a critical livelihood source for bivalve harvesters, yet increasing anthropogenic activities threaten the safety of its aquatic food sources. This study comprehensively examines the microbial communities associated with freshwater bivalves (*Batissa violacea*) and river sediments, focusing on antibiotic resistance and heavy metal (HM) tolerance. Samples from soft tissues, gills, and sediments were subjected to Most Probable Number (MPN) tests, antibiotic susceptibility assays, HM tolerance analysis, and 16S rRNA gene sequencing to identify microbial taxa and their functional characteristics. Several pathogenic bacteria were isolated and identified, including *Vibrio cholerae*, *Vibrio parahaemolyticus*, *Salmonella* spp., and thermotolerant *Escherichia coli*. Most isolates exhibited multidrug resistance, particularly against rifampicin, vancomycin, penicillin, streptomycin, and tetracycline, posing potential risks to public health and food safety. Conversely, HM tolerance analysis revealed limited microbial resistance to zinc, copper, cadmium, chromium, and lead, predominantly at concentrations of 1 mM. The presence of these antibiotic-resistant and HM-tolerant microbes underscores the critical interplay between environmental contamination and microbial evolution, presenting challenges for both ecosystem health and human consumption safety. The findings highlight the urgent need for integrated environmental management and interventions to mitigate microbial contamination in aquatic systems.

## Importance

The Ba River in Fiji is a vital resource for communities dependent on “kai” (freshwater bivalve) harvesting. However, industrial, agricultural, and domestic waste discharge increasingly contaminate the river, raising concerns about the safety of its aquatic resources. This study investigated the microbial communities associated with these bivalves and the river sediments, specifically examining their resistance to antibiotics and tolerance to heavy metals. The findings revealed the presence of several pathogenic bacteria, including *Vibrio* and *Salmonella* species, a significant proportion of which exhibited resistance to commonly used antibiotics. While some microbial tolerance to heavy metals such as copper and lead was observed, it remained generally limited. These results suggest that environmental pollution is likely contributing to the spread of antibiotic-resistant and potentially harmful microorganisms, thereby posing a risk to the ecological health of the river, the bivalve populations, and the well-being of the local communities that rely upon them.

## Introduction

In Pacific Island countries (PICs), where seafood is foundational to both diet and culture, bivalves represent a primary food source, especially in Fiji. While bivalve production has grown substantially [1], aquatic environments are increasingly strained by anthropogenic pollution [2]. Rising faecal contamination, in particular, poses a serious threat to food safety [3,4]. As filter feeders, bivalves process vast quantities of water, leading to bioaccumulation of faecal pathogens [5], such as *Escherichia coli*, Faecal *Streptococci*, and *Klebsiella* spp., which can cause acute intestinal illnesses [6]. Consequently, *E. coli* and faecal coliforms serve as standard indicators for sanitary quality seafood and water [7,8]. Given that bivalves are traditionally consumed raw or lightly cooked with viscera intact, they represent a high-risk vector for outbreaks of typhoid fever, cholera, and hepatitis [9], often linked to *Vibrio* species [10,11]. Beyond microbial threats, bivalve safety is further undermined by chemical pollutants, specifically heavy metals. While naturally present in trace amounts, metal concentrations have surged due to mining, agricultural runoff, and industrial waste discharge [12,13]. These metals persist in the environment and bioaccumulate within the food chain and concentrate in bivalve tissues, posing long-term health risks to human consumers [14–17]. Interestingly, several bacteria have acquired metal resistance genes (MRGs) to survive heavy metal exposure, allowing them to tolerate and even flourish in these toxic conditions [18]. Similarly, antimicrobial resistance (AMR) is driven by the extensive use and misuse of antibiotics, leading to the rise of microbes that are resistant to current prescribed antibiotics [19]. Recent studies reveal a compelling correlation between metal tolerance and antibiotic resistance through a process known as co-selection [20,21]. This is attributed to the fact that the metal resistance genes (MRGs) and the antibiotic-resistant genes (ARGs) are often located on the same mobile genetic elements (plasmids, integrons, and transposons), so heavy metal pressure can drive the persistence and horizontal transfer of AMR, even in the absence of antibiotics [21,22]. This phenomenon significantly accelerates the spread of multi-drug resistance pathogens in contaminated aquatic environments, presenting a complex and escalating challenge to global public health.

*Batissa violacea*, known locally as “kai”, is Fiji`s only known freshwater bivalve. Harvested from the major rivers, it serves as a crucial protein source, a rich supplier of essential minerals (including calcium, potassium, zinc, and iron) [23], and a vital economic asset for local communities [24]. The freshwater *kai* fishery is a key contributor to Fijian households and the local economy, with annual market sales reaching an estimated 2,526 tonnes, generating approximately FJD $2.2 million [25]. Despite its socio-economic importance, research on the microbial safety of freshwater bivalves in the Pacific region, particularly in Fiji, remains significantly limited. This study addresses this critical data gap by investigating the microbial communities associated with *Batissa violacea* and its surrounding sediments in the Ba River. By integrating traditional culture-based methods with high-resolution 16S rRNA gene sequencing, this research ensures precise taxonomic identification of the resident bacteria. Furthermore, by comparing bacterial concentrations across the bivalve`s soft tissues, gills, and river sediments, this study offers a comprehensive understanding of the contamination pathway. Finally, we evaluate the antibiotic resistance and heavy metal tolerance profiles of the isolated strains, highlighting the potential public health risks for local communities that rely on these bivalves as a primary food source.

## Materials and methods

### Sample collection

#### Sites.

**Fig 1:**
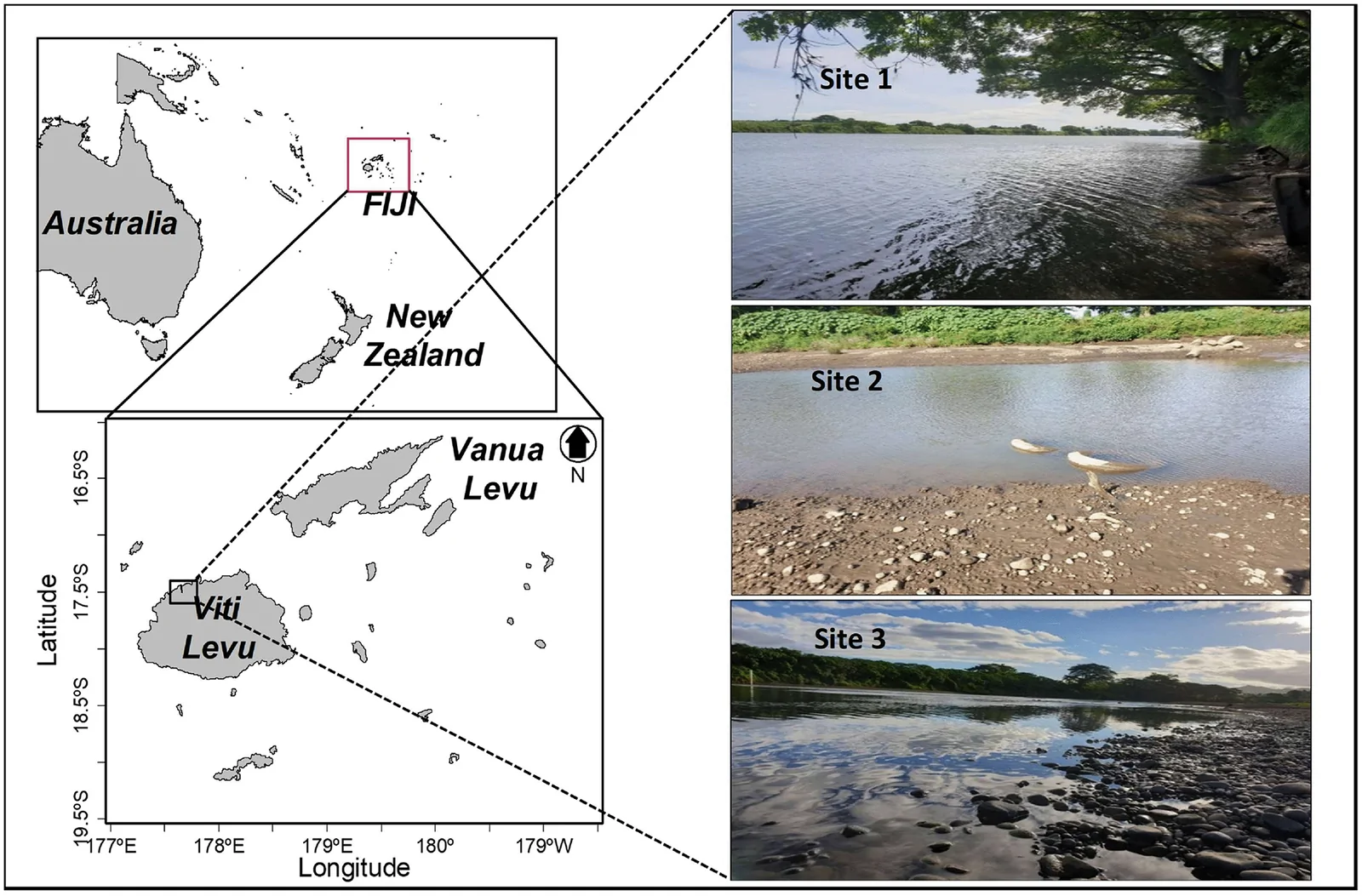
Map of sampling sites location for collection of bivalves (*Batissa violacea*) and sediment samples along the Ba River, situated in the Ba province in the north-eastern section of the main Island of Viti Levu in Fiji. Site 1 is behind the Rarawai Sugar Mill and near Ba town, Site 2 is further downstream, and Site 3 is a supposedly pristine site, upstream of Rarawai mill and Ba town. (*Photo credit: Divya Darshika Mala*).

This study was conducted along the Ba River, located in the Western Division on the main Island of Viti Levu, Fiji (Table 1). The river flows through Ba town and has a total length of approximately 22 km and a maximum water depth of 8 m [26]. Traditional local protocol (*sevusevu*) was followed to obtain sampling permission from local communities that own and manage the river. Three sites were selected for sample collection (Fig 1). Site 1 was situated 1.8 km upstream from Site 2 and was located behind the Rarawai sugar mill. This site was selected to assess the impact of industrial waste deposition. Site 2, located under the Yalalevu Bridge, was chosen due to its proximity to Ba town and being a common place for harvesting *B. violacea* (kai). Site 3 was situated 4.8 km upstream from Site 1 at the Vaqia River, selected as a control or pristine environment due to its distance from urban and industrial influences. The samples were collected from three distinct sites to capture environmental variability associated with upstream, downstream, and effluent-impacted conditions.

**Table 1:** Physiochemical properties and sediment and water samples from different sites and location description. TL – Top left, TR – Top right, C – Center, BL – Bottom left, BR – Bottom right.

Site	Position	GPS	Elevation (m)	Sediment		Water		Site Description
				Temp (°C)	pH	Temp (°C)	pH	
**1**	**TL**	17^o^3235.8 S, 177^o^4053.0 E	6	26.1	6.88	26.2	7.11	Metals deposited at the river bank Sugar mill waste pipe located at the mouth of the river bank Black peat soil
	**TR**	17^o^3235.7 S, 177^o^4053.0 E	7	26.8	6.73	26.2	7.11	
	**C**	17^o^3235.9 S, 177^o^4053.1 E	4	26.2	6.84	26.2	7.11	
	**BL**	17^o^3235.8 S, 177^o^5053.9 E	6	27.2	6.90	26.2	7.11	
	**BR**	17^o^3238.8 S, 177^o^4053.1 E	10	24.4	6.81	26.2	7.11	
**2**	**TL**	17^o^3212.3 S, 177^o^4055.9 E	5	27.0	7.16	26.6	7.11	Waste deposits such as old tyres, Styrofoam boxes and plastic bags near the river bank Black silt soil
	**TR**	17^o^3212.7 S, 177^o^4055.5 E	5	27.0	7.16	26.6	7.11	
	**C**	17^o^3212.3 S, 177^o^4056.0 E	4	27.3	6.92	26.6	7.21	
	**BL**	17^o^3212.4 S, 177^o^4055.9 E	4	27.0	6.95	26.6	7.17	
	**BR**	17^o^3212.4 S, 177^o^4056.0 E	4	27.0	7.07	26.5	7.2	
**3**	**TL**	17^o^3438.7 S, 177^o^4127.9 E	3	27.6	7.26	26.2	7.29	Second most common bivalve collection site in Ba Black silt soil
	**TR**	17^o^3238.7 S, 177^o^4127.8 E	5	26.08	7.05	25.9	7.34	
	**C**	17^o^3438.7 S, 177^o^4127.8 E	2	26.2	7.09	25.9	7.18	
	**BL**	17^o^3438.7 S, 177^o^4127.9 E	4	26.0	7.26	26.9	7.16	
	**BR**	17 ^o^3438.7 S, 1774127.8 E	1	26.6	6.98	26.0	7.38	

#### Sampling.

A total of 90 bivalve samples (30 bivalves per site) and 15 sediment samples (5 pool samples per site) were collected. This sample size allowed the detection of consistent microbial patterns across sites while minimizing sampling bias. All samples were transported to the laboratory in an ice box within 5 hrs of collection and preserved at −20 ˚C until required. All aseptic techniques were strictly followed during the entire sample collection to prevent cross contamination. Sediment samples were collected using 10 m × 10 m quadrat. About 100 g of sediment sample was collected from the four corners and the centre using a grab sampler. The samples from each site were pooled for microbiological study [27]. Physical parameters, including pH and temperature of both water and sediment samples were measured with standard procedure and recorded *in situ* for each respective site (Table 1).

### Bacteriological study

#### Aerobic bacteria.

Bivalve samples (*n* = 10) of similar size (5–9 cm shell length) were aseptically dissected to extract soft tissues and gills. A 10 g of each tissue sample (soft tissue and gill) was homogenized in 90 mL of sterilized phosphate buffer (PB; pH 7.0) using a homogenizer (BioLab, New Zealand), resulting in a 10 ^−^ ¹ dilution. The homogenate was serially diluted up to 10 ^−^ ⁷ using 9 mL of sterile PB. From each dilution, 100 µL was plated on plate count agar (PCA; Difco, USA) using the spread plate method and incubated at 37 °C for 24–48 hrs. For sediment samples, 10 g of each pooled sediment was mixed with 90 mL of sterilized distilled water to create a 10 ^−^ ¹ dilution, which was then serially diluted up to 10 ^−^ ⁷ using 9 mL of sterile distilled water. From each dilution, 1 mL aliquot was plated onto the nutrient agar (NA; Difco, USA) using the pour plate method. All Petri dishes were incubated at 37 °C for 24–48 hrs. The results obtained were expressed as the number of colony-forming units per gram (CFU/g) of the sample [7]. A total of 117 distinct colonies were isolated from the soft tissues, gills, and sediments based on their unique morphological characteristics. All colonies were purified on nutrient agar (NA; Difco, USA) and stored on sterile NA slants at −8°C for subsequent analysis.

### Isolation of bacterial pathogens

Serially diluted suspensions derived from bivalve tissue and sediment samples were utilized to isolate specific human pathogenic bacteria. Seven selective media were employed to enumerate and tentatively identify *Vibrio* spp., *Salmonella* spp., *Shigella* spp., *Streptococcus* spp., *Klebsiella* spp., coliforms, and faecal coliforms. The media included Thiosulfate-Citrate-Bile Salts-Sucrose (TCBS) agar, Xylose Lysine Deoxycholate (XLD) agar, KF Streptococcal agar, MacConkey agar, M-Endo agar, Eosin Methylene Blue (EMB) agar, and m-Faecal Coliform (MFC) agar. Pathogens isolated from these primary cultures were further subjected to Gram staining and microscopic observation following the methodology outlined by Smith and Hussey [28].

### Coliform count

Bivalve tissue homogenate was used for coliform enumeration. A 1 mL sample was inoculated into a 10 mL sterile Lauryl Tryptose Broth (LTB) fitted with an inverted Durham tube. The tube was incubated at 37 °C for 24 hrs and checked for gas production (presumptive test). Tubes that showed gas production (positive result) were inoculated (1 mL) into 10 mL of Brilliant Green Lactose Broth (BGLB) and incubated at 37 °C for 24 hrs (confirmatory test). The gas-positive tubes were recorded to determine the Most Portable Number (MNP) index using a standardized MPN table [29]. The positive BGLB tubes were streaked onto EMB agar plates and incubated at 37 °C and 44.5 °C for 24–48 hrs to distinguish the *E. coli* from the thermo-tolerant *E. coli*. All bacteria analyses were conducted in triplicate, the mean values were calculated and used for statistical analysis.

### DNA extraction and 16s rRNA gene PCR sequencing

Thirty aerobic bacterial isolates were selected for 16S rRNA gene PCR sequencing. Further, bacteria were exclusively selected to facilitate functional studies such as testing antibiotic resistance or tolerance to heavy metals. DNA was extracted from the bacterial isolates following the MicroGEM bacterial extraction kit protocol (https://microgembio.com/product/prepgem-bacteria-dna-extraction-kit/). The 16S rRNA gene was amplified using universal primers 27F (AGAGTTTGATCMTGGCTCAG) and 1492R (CGGTTACCTTGTTACGACTT). Purified PCR products were sequenced using the ABI Prism Sequencer 3730 (Applied Biosystems, USA). Chimera analysis of the 16S rRNA gene sequences was performed using Bellerophon software. The sequences were then compared against the eubacteria RefSeq Genome database using BLASTn to identify closely related type strains. Multiple sequence alignment was carried out with MUSCLE, as previously described by Pipite [30].

### Heavy metal tolerance test

All isolated heterotrophic bacterial strains were evaluated for heavy metal tolerance using the agar dilution method. This established approach allows for the simultaneous evaluation of multiple isolates against defined concentrations of antibiotics or heavy metals under controlled conditions. Each strain was individually inoculated into 10 mL of sterile nutrient agar medium supplemented with varying concentrations (1 mM, 3 mM, 5 mM, and 10 mM) of selected heavy metals. Due to Site 1’s proximity to a known effluent discharge from the sugar mill, it is likely to experience elevated levels of pollutants, including heavy metals. In this regard, the selected concentration range of heavy metals (1–10 mM) was used to assess bacterial tolerance and adaptive responses to metal stress. Zn, Cu, Cd, Cr and Pb were chosen for analysis because these metals were found at high concentrations in bivalve soft tissues from Ba River. The cultures were incubated at 37 °C for 24–28 hrs. Each Petri plate was divided into six sections, and 10 *µ*L of each bacterial strain was spot inoculated onto the agar surface. Control plates with streaked bacterial strains and no heavy metals were prepared for comparison. All experiments were conducted in triplicate to ensure reproducibility.

### Antibiotics sensitivity test

The panel of antibiotics (streptomycin, penicillin, vancomycin, rifampicin, and tetracycline) was selected to represent a range of mechanisms of action, including inhibition of cell wall synthesis, protein synthesis, and RNA transcription, allowing for a comprehensive assessment of bacterial susceptibility and resistance pattern [31]. Isolated bacterial strains were individually inoculated into 10 mL of sterile nutrient agar medium, supplemented with concentrations of 25 *µ*g, 50 *µ*g, 75 *µ*g, and 100 *µ*g of each antibiotic. The cultures were incubated at 37 °C for 24–28 hrs. Each Petri plate was divided into six sections, and 10 *µ*L of each bacterial strain was spot-inoculated onto the agar surface. The control plates without the antibiotics, were streaked with bacterial strains and incubated under the same conditions at 37 °C for 24–48 hrs for comparison.

### Statistical analysis

Microbial samples were analysed using single factor ANOVA. Significant results were subjected to Tukey HSD post hoc pairwise comparison test. Experimental data were collected in triplicates. All data were subjected to exploratory analysis to identify any irregularities and bias including outliers. Variables were then subjected to normality and homogeneity of variance assumptions tests as conditions for using one way ANOVA. Alpha level of *p* < 0.05 was selected to determine acceptable significance. Statistical analysis was performed using R version 4.3.2 [32].

## Results

The socioeconomic livelihoods of communities around the Ba watershed in Fiji are significantly dependent on its water and food resources, particularly the harvesting of kai bivalves for consumption and sale. Due to increasing concerns regarding the degradation of these aquatic resources, this study conducted a thorough examination of the microbial communities associated with the bivalves and river sediments. The investigation focused on microbial characterization, antibiotic sensitivity and tolerance, and heavy metal tolerance.

**Isolation and enumeration of aerobic bacteria from soft tissues, gills and sediments**

**Fig 2:**
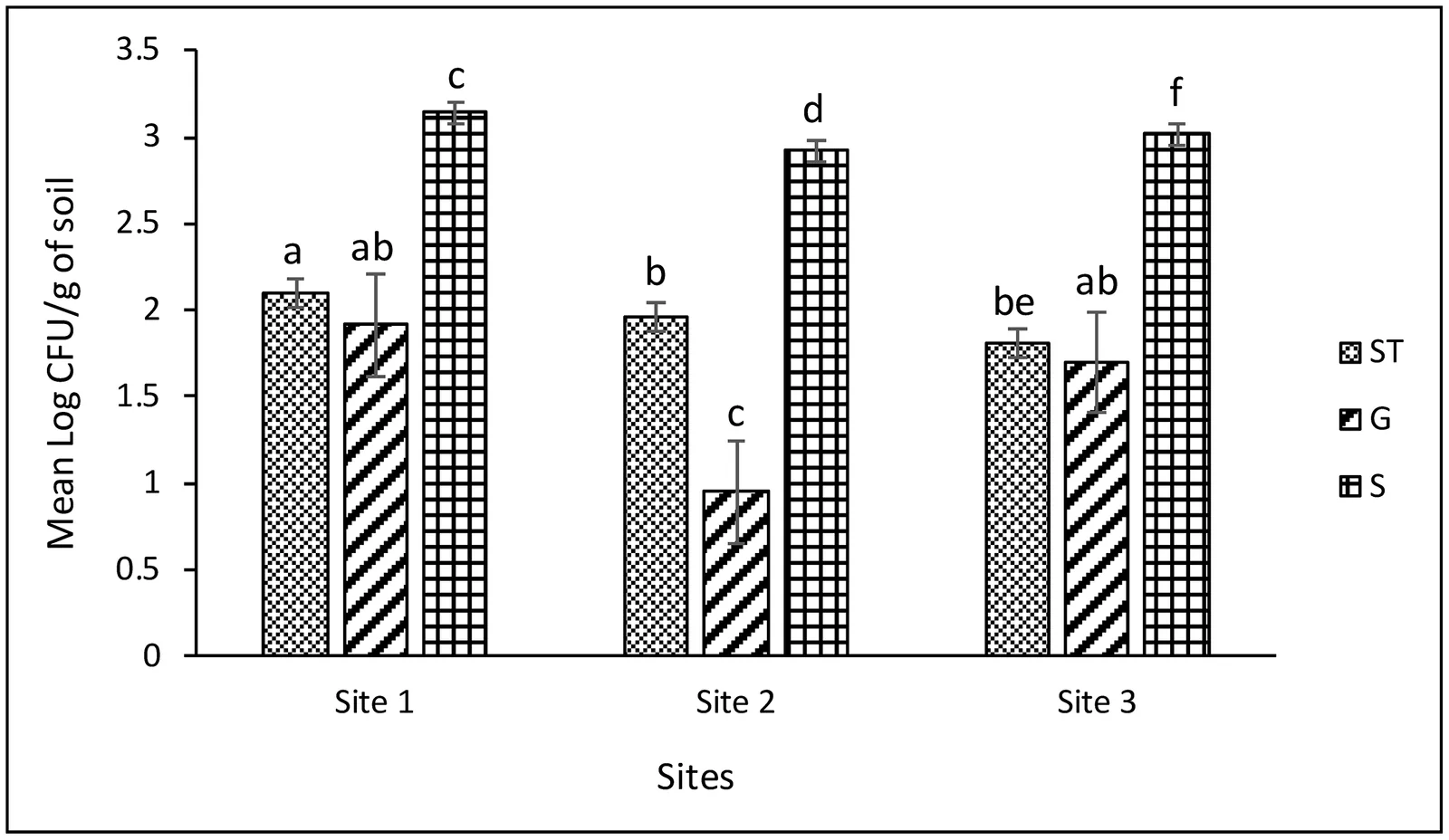
Total aerobic bacteria load in soft tissues (ST) and gills (G) of bivalves (*Batissa violacea*) and sediments (S) at different sites along the Ba River in Fiji. Different lowercase letters (a-f) denote significant differences between total microbial abundance in soft tissues, gills and sediments at 3 sites (ANOVA with Tukey HSD pairwise comparisons, *p* < 0.05). Vertical bars represent ± S.E. of the mean.

The aerobic bacteria were isolated from soft tissues, gills, and sediments. In bivalves, the maximum bacterial load was observed in the soft tissues (ranging from 7.9 × 10^3^ cfu/g to 2.2 × 10^4^ cfu/g), followed by the gills (3.4 × 10^3^ cfu/g–1.4 × 10^4^ cfu/g). Conversely, sediments recorded the lowest bacterial count (2.6 × 10^4^ cfu/g to 2.9 × 10^4^ cfu/g) across all sites. Statistical analysis indicated significant differences (*p* < 0.05, F–value = 1.95) in bacterial load among the soft tissues, gills, and sediments at each site (Fig 2). Site 1 appeared to be most heavily contaminated, exhibiting the highest aerobic bacterial loads compared to Sites 2 and 3. Site 3 showed higher bacterial loads in soft tissues and sediments than Site 2. However, these results may vary based on the season, sampling location, and bivalves’ species.

### Human pathogenic bacteria

***Vibrio* spp. isolation and enumeration in bivalves’ soft tissues, gills and sediments**

**Fig 3:**
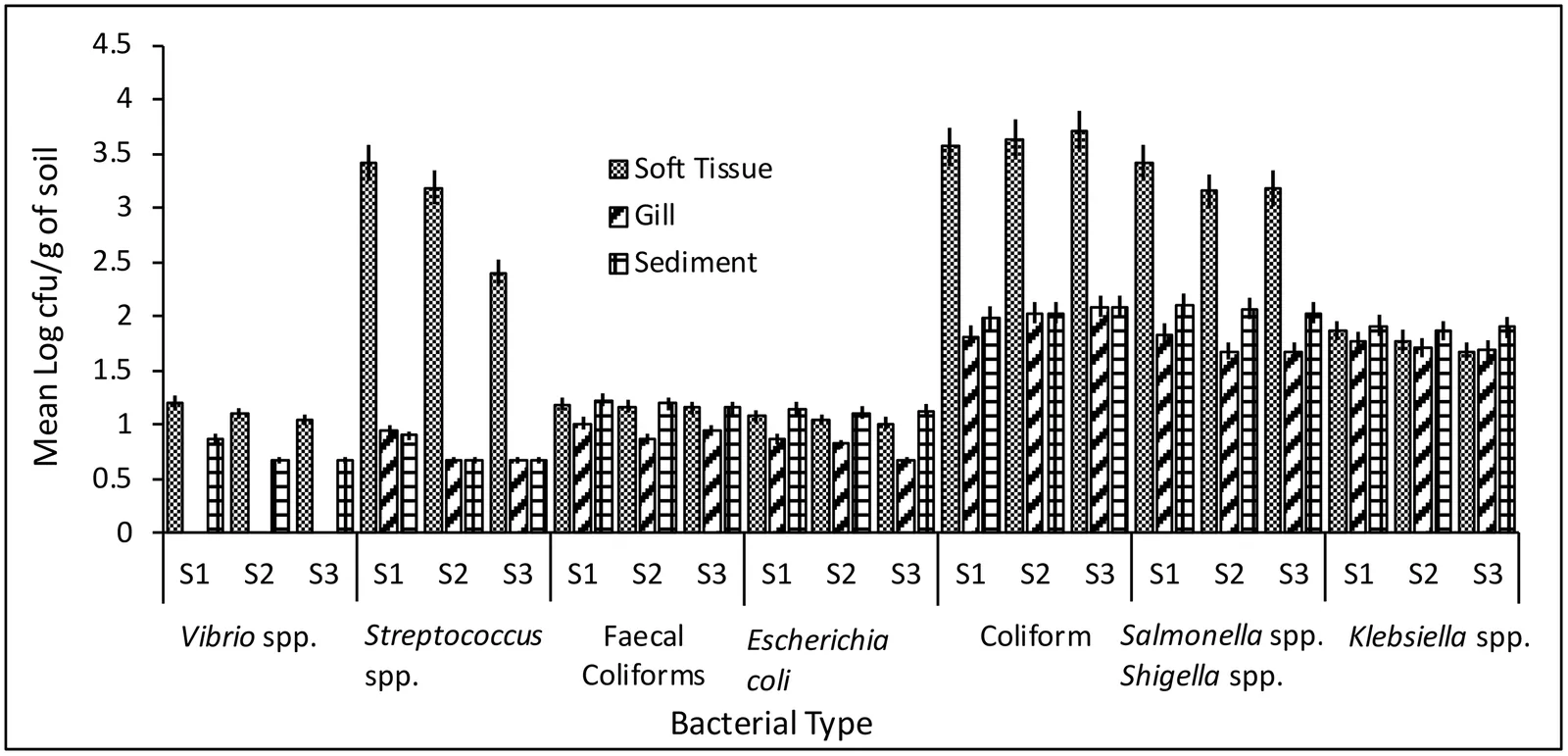
Various human pathogenic bacteria in soft tissues and gills of bivalves (*Batissa violacea*) and sediments from the three sites. S1 – Site 1, S2 – Site 2, S3 – Site 3 along the Ba river in Fiji. Values are presented as means ± standard deviation of triplicate samples.

Among the soft tissues, gills, and sediments, soft tissues recorded the highest bacterial load of *Vibrio* spp. at Site 1 (4.0 × 10^3^ cfu/g). While *Vibrio* spp. was present in the soft tissues and sediments, no growth was observed in the gill samples. Statistical analysis showed no significant difference (*p* > 0.05) in *Vibrio* spp. bacterial loads amongst various sites (Fig 3).

**Faecal *Streptococcus* isolation and enumeration in bivalves’ soft tissues, gills and sediments**

Faecal *Streptococcus* was most prevalent in the soft tissues across all sites, with loads ranging from 1.0 × 10^2^ cfu/g to 2.7 × 10^4^ cfu/g. In contrast, gills and sediments recorded lower bacterial loads of 7.0 × 10^2^ cfu/g and 5.0 × 10^2^ cfu/g, respectively. These results indicate that bivalves from Site 1 were heavily contaminated with faecal matter, exhibiting the highest load of faecal *Streptococcus*, followed by Sites 2 and 3. Statistical analysis confirmed a significant difference (*p* < 0.05, F–value = 186.9) in the bacterial loads of soft tissues across the different sites (Fig 3).

**Faecal coliforms isolation and enumeration in bivalves’ soft tissues, gills and sediments**

Notably, the presence of faecal coliforms in both bivalves and sediments of the Ba River further confirms faecal contamination in the aquatic environments. Statistical analysis showed a significant difference (*p* < 0.05, F–value = 13.34) in the faecal coliform bacterial loads across different sampling sites.

***Escherichia coli* isolation and enumeration in bivalves’ soft tissues, gills and sediments**

The maximum *E. coli* populations were observed in the soft tissues (4.0 × 10^2^ cfu/g), sediments (2.7 × 10^2^ cfu/g), and gills (1.7 × 10^2^ cfu/g). Despite these findings, statistical analysis indicated no significant difference (*p* > 0.05) in the bacterial load at different sampling sites (refer to Fig 3).

**Coliforms isolation and enumeration in bivalves’ soft tissues, gills and sediments**

Coliform levels were analysed in the soft tissues, gills and sediments of the Ba River. Soft tissues recorded the highest coliform count (2.9 × 10^4^ cfu/g), while the lowest count was recorded in the gills (1.2 × 10^4^ cfu/g). Statistical analysis showed no significant difference in coliform levels between the study sites (*p* > 0.05).

***Salmonella* spp. and *Shigella* spp. isolation and enumeration in bivalves’ soft tissues, gills and sediments**

*Salmonella* spp. and *Shigella* spp. were detected in the soft tissues, gills, and sediments. The soft tissues exhibited the highest microbial load, followed by the sediments, while the lowest load was observed in the gills.

***Klebsiella* spp. isolation and enumeration in bivalves’ Soft tissues, gills and sediments**

The results demonstrated that soft tissues contained a higher bacterial load of *Klebsiella* spp. (9.9 × 10^4^ cfu/g) compared to the gills (2.8 × 10^3^ cfu/g) and sediments (1.1 × 10^4^ cfu/g). However, statistical analysis showed no significant difference in bacterial load among these three samples at any sites (*p* > 0.05) (Fig 3).

### Enumeration of coliforms using most probable number method

The Most Probable Number (MPN) method was conducted in three stages: the presumptive, confirmatory, and complete tests. Initially, the presumptive test was carried out on the soft tissues, gills, and sediments. All five tubes for each sample type showed positive results and were subsequently selected for confirmatory testing. The confirmatory test also resulted in five positive tubes for all samples, confirming the presence of coliforms (Table 2). These results were compared against the Food and Drug Administration’s Bacterial Analytical Manual [33] standards, confirming that the soft tissues, gills and sediments contained more than 1600 MPN of coliforms per 100 g. The completed MPN test identified the presence of thermotolerant *E*. *coli* in soft tissues and sediments across all sites. While gills from Site 1 showed no presence of thermotolerant *E*. *coli*, the remaining two sites reported positive results for the bacteria in gill samples.

**Table 2:** Complete MPN test for soft tissues, gills and sediments.

	S1			S2			S3		
	Single Strand		Double Strand (10 ml)	Single Strand		Double Strand (10 ml)	Single Strand		Double Strand (10 ml)
	0.1 ml	1ml		0.1 ml	1ml		0.1 ml	1ml	
**ST**	**+**	**+**	**+**	**+**	**+**	**+**	**–**	**+**	**+**
**G**	**–**	**–**	**–**	**+**	**+**	**+**	**–**	**+**	**+**
**S**	**+**	**+**	**+**	**+**	**+**	**+**	**+**	**+**	**+**

* **+** Growth at 44.5 °C; **–** No growth at 44.5 °C

### Isolation of selected bacterial strains

Nutrient Agar media was used to isolate bacterial strains *via* the streak plate method [34]. A total of 117 colonies were selected from the soft tissues, gills and sediments; of these, 53 were identified as aerobic bacteria and 64 as human pathogens. The identified pathogenic strains are detailed in Table 3. Thirty of the aerobic bacteria were selected for molecular analysis based on their unique morphological characteristics. Most colonies were circular, flat, mucoid, and possessed entire margins. In addition, all 117 colonies were subjected to heavy metal tolerance and antibiotic resistance testing.

**Table 3:** Human pathogen colonies in soft tissues, gills and sediments of bivalves from different sites.

Bacterial Strain	Colony Size	Color/ Pigment	Form	Margin	Elevation	Other Features	Name
**S1ST022**	Medium	Yellow	Circular	Entire	Raised	Mucoid	Coliforms
**S1ST023**	Medium	Red	Circular	Entire	Raised	Mucoid	*Shigella* spp.
**S1ST024**	Small	Red	Circular	Entire	Flat	Mucoid	*Streptococcus* spp.
**S1ST025**	Medium	Pale pink	Circular	Entire	Flat	Mucoid	*Enterococcus* spp.
**S2ST026**	Medium	Yellow	Circular	Entire	Raised	Mucoid	Coliforms
**S2ST027**	Medium	Red	Circular	Entire	Raised	Mucoid	*Shigella* spp.
**S2ST028**	Small	Red	Circular	Entire	Flat	Mucoid	*Streptococcus* spp.
**S2ST029**	Medium	Pink	Circular	Entire	Raised	Mucoid	*Klebsiella* spp.
**S3ST030**	Small	Yellow	Circular	Entire	Raised	Mucoid	Coliforms
**S3ST031**	Medium	Black	Circular	Entire	Flat	Mucoid	*Salmonella* spp.
**S3ST032**	Medium	Red	Circular	Entire	Raised	Mucoid	*Shigella* spp.
**S3ST033**	Small	Red	Circular	Entire	Flat	Mucoid	*Streptococcus* spp.
**S3ST034**	Minute	Pink	Circular	Entire	Raised	Mucoid	Coliforms
**S3ST035**	Minute	Pink	Circular	Entire	Flat	Mucoid	Coliforms
**S3ST036**	Medium	Yellow	Circular	Entire	Flat	Mucoid	*Vibrio* spp.
**S3ST037**	Small	Yellow	Circular	Entire	Flat	Mucoid	*Vibrio* spp.
**S3ST038**	Medium	Green	Circular	Entire	Flat	Mucoid	*Vibrio* spp.
**S3ST039**	Small	Yellow	Circular	Entire	Flat	Mucoid	*Vibrio* spp.
**S3ST040**	Small	Green	Circular	Entire	Flat	Mucoid	*Vibrio* spp.
**S3ST041**	Large	Yellow	Circular	Entire	Flat	Mucoid	*Vibrio* spp.
**S3ST042**	Small	Yellow	Circular	Entire	Flat	Mucoid	*Vibrio* spp.
**S3ST043**	Small	Yellow	Circular	Entire	Flat	Mucoid	*Vibrio* spp.
**S3ST044**	Small	Green	Circular	Entire	Flat	Mucoid	*Vibrio* spp.
**S3ST045**	Medium	Green	Circular	Entire	Flat	Mucoid	*Vibrio* spp.
**S1ST046**	Small	Green	Circular	Entire	Flat	Metallic Sheen	Thermotolerant *E.coli*
**S2ST047**	Small	Green	Circular	Entire	Flat	Metallic Sheen	Thermotolerant *E.coli*
**S3ST048**	Small	Green	Circular	Entire	Flat	Metallic Sheen	Thermotolerant *E.coli*
**S1G021**	Small	Yellow	Circular	Entire	Raised	Mucoid	Coliforms
**S1G022**	Medium	Black	Circular	Entire	Flat	Mucoid	*Salmonella* spp.
**S1G023**	Pin point	Red	Circular	Entire	Raised	Mucoid	*Shigella* spp.
**S1G024**	Pin point	Pale pink	Circular	Entire	Flat	Mucoid	*Enterococcus* spp.
**S1G025**	Pin point	Colorless	Circular	Entire	Flat	Mucoid	Coliforms
**S2G026**	Medium	Red	Circular	Entire	Flat	Mucoid	*Streptococcus* spp.
**S2G027**	Pin point	Red	Circular	Entire	Raised	Mucoid	*Shigella* spp.
**S2G028**	Medium	Yellow	Circular	Entire	Raised	Mucoid	Coliforms
**S3G029**	Pin point	Red	Circular	Entire	Flat	Mucoid	*Streptococcus* spp.
**S3G030**	Pin point	Pale pink	Circular	Entire	Flat	Mucoid	*Enterococcus* spp.
**S3G031**	Medium	Black	Circular	Entire	Flat	Mucoid	Coliforms
**S3G032**	Medium	Red	Circular	Entire	Flat	Mucoid	*Streptococcus* spp.
**S3G033**	Pin point	Black	Circular	Entire	Flat	Mucoid	Coliforms
**S1G034**	Small	Green	Circular	Entire	Flat	Metallic Sheen	Thermotolerant *E.coli*
**S2G035**	Small	Green	Circular	Entire	Flat	Metallic Sheen	Thermotolerant *E.coli*
**S3G036**	Small	Green	Circular	Entire	Flat	Metallic Sheen	Thermotolerant *E.coli*
**S1S022**	Pin point	Red	Circular	Entire	Raised	Mucoid	*Shigella* spp.
**S1S023**	Small	Pink	Circular	Entire	Raised	Mucoid	*Klebsiella* spp.
**S1S024**	Medium	Pink	Circular	Entire	Raised	Mucoid	Coliforms
**S1S026**	Small	Pink	Circular	Entire	Raised	Mucoid	Coliforms
**S1S027**	Medium	Yellow	Circular	Entire	Flat	Mucoid	*Vibrio* spp.
**S1S028**	Medium	Green	Circular	Entire	Flat	Mucoid	*Vibrio* spp.
**S2S029**	Small	Pink	Circular	Entire	Raised	Mucoid	*Klebsiella* spp.
**S2S031**	Small	Green	Circular	Entire	Flat	Metallic Sheen	*E.coli*
**S2S032**	Small	Pink	Circular	Entire	Raised	Mucoid	Coliforms
**S2S033**	Small	Green	Circular	Entire	Flat	Metallic Sheen	Coliforms
**S2S034**	Small	Yellow	Circular	Entire	Flat	Mucoid	*Vibrio* spp.
**S3S035**	Small	Green	Circular	Entire	Flat	Mucoid	*Vibrio* spp.
**S3S036**	Pin point	Red	Circular	Entire	Raised	Mucoid	*Shigella* spp.
**S3S037**	Small	Pink	Circular	Entire	Raised	Mucoid	*Klebsiella* spp.
**S3S038**	Small	Green	Circular	Entire	Flat	Metallic Sheen	*E.coli*
**S3S039**	Small	Green	Circular	Entire	Flat	Metallic Sheen	Coliforms
**S3S040**	Small	Yellow	Circular	Entire	Flat	Mucoid	*Vibrio* spp.
**S3S041**	Small	Green	Circular	Entire	Flat	Mucoid	*Vibrio* spp.
**S1S042**	Small	Green	Circular	Entire	Flat	Metallic Sheen	Thermotolerant *E.coli*
**S2S043**	Small	Green	Circular	Entire	Flat	Metallic Sheen	Thermotolerant *E.coli*
**S3S044**	Small	Green	Circular	Entire	Flat	Metallic Sheen	Thermotolerant *E.coli*

* S1, S2, S3- Sites; ST- Soft Tissues; G- Gills; S- Sediments; 005, 006 etc. Strain number.

### 16S rRNA gene sequencing for aerobic bacteria

**Fig 4:**
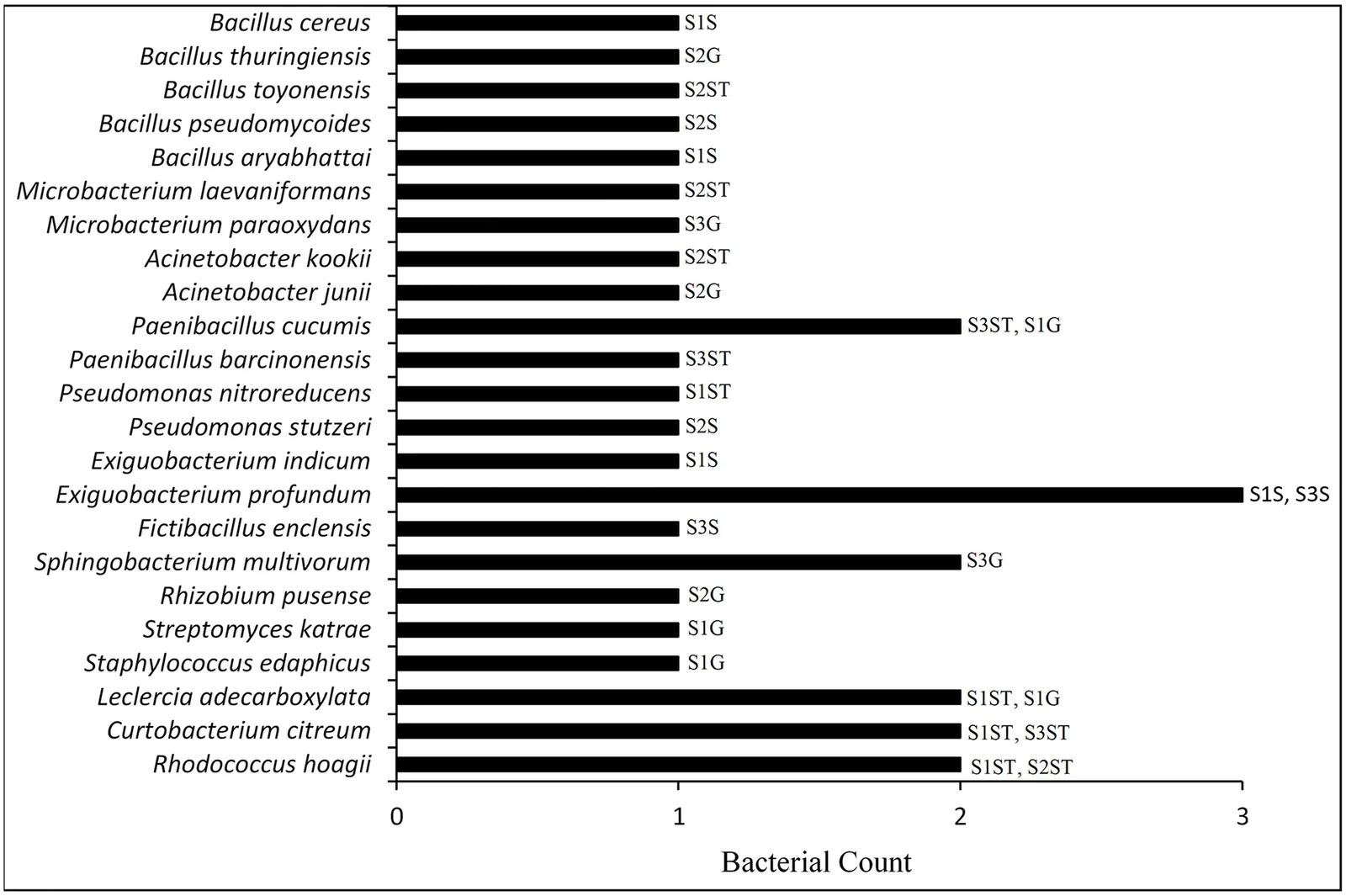
Bacteria identified by 16s rRNA gene sequencing of bivalve and sediment samples from different sites along the Ba in Fiji. The x-axis shows the frequency of occurrence of each bacteria species in the samples (bivalves and sediments). S1, S2, S3- Sites; ST- Soft Tissues; G- Gills; S- Sediments.

16s rRNA Gene sequencing was utilized to identify bacteria isolated from the soft tissues, gills, and sediments. Fig 4 shows the results of the 16s rRNA PCR sequencing for these strains. The sequencing focused specifically on the isolated bacterial cultures; of the 30 bacterial strains analysed, 23 distinct bacterial species were identified. Detailed descriptions of each identified strain are provided in Table 4.

**Table 4:** 16s rRNA bacterial strain description.

Bacteria	Description	Reference
***Rhodococcus hoagii***	Formely known as *Rhodococcus equi,* Gram positive bacteria, Soil dwelling actinomycete, grows well at temperatures ranging from 10° to 40° C, Human and animal pathogen (mostly horses and pigs), Causes pneumonia in foals, Affects immunosuppressed humans such as AIDS patient	[35,36]
***Curtobacterium citreum***	Aerobic, non-spore forming, Gram-positive bacillus, has been isolated from plants but not a plant pathogen, have not been any reports of human infections	[37]
***Leclercia adecarboxylata***	Gram negative rods, Distributed widely in food and water, a part of normal flora in the gut of animals and in the stool of humans, Extremely rare human pathogen, Most commonly affecting immune compromised individuals, such as cancer, leukemia, renal failure and cirrhosis, Susceptible to many anti-biotics but multidrug resistant strains have been reported recently	[38]
***Pseudomonas nitroreducens***	Gram-negative, rod-shaped soil bacteria, Psychrotrophic bacterium, Ability to synthesize polyhydroxybutyrate (PHB) and could be used to produce biodegradable plastic	[39]
***Bacillus toyonensis***	Gram positive bacteria, Bioflocculant producing bacteria, naturally occurring in soils, non-toxigenic and non-pathogenic, Optimum growth at 35 °C, Used as a probiotic in animal feed, Resistant to chloramphenicol and tetracycline	[40]
***Microbacterium laevaniformans***	Gram positive, Occur in raw sewage and in activated sludge, Tolerant to heavy metals (uranium, nickel, cobalt and cadmium)	[41]
***Acinetobacter kookii***	Gram negative, Novel bacteria isolated from soil, Grows over a broad range of temperatures (24–41 °C)	[42]
***Paenibacillus barcinonensis***	Isolated from a rice field, Gram positive, Growth occurs at temperatures in the range 10–40 °C and at pH 5–10.4	[43]
***Microbacterium natoriense***	Novel sp. isolated from soil in Natori, Japan, Gram-positive, rod-shaped bacteria, Optimum temperature for growth 30 °C, pH optimum for growth of 5–7, Produces D-aminoacylase (enzyme function in xenobiotic metabolism)	[44]
***Staphylococcus edaphicus***	Novel sp. isolated in Antarctica, Gram positive, spherical or irregular cocci bacteria, Growth temperature ranging from 10 °C- 42 °C, Antimicrobial resistance genes were found in the genome	[45]
***Streptomyces katrae***	Isolated from soil in India, Gram-positive filamentous bacteria, Highly sensitive to amikacin, ampicillin, ciprofloxacin, erythromycin, gentamicin, imipenem, norfloxacin, tetracycline, and vancomycin. Resistance towards chloramphenicol, cephalothin, cephoxitin, co-trimoxazole, oxacillin, penicillin, piperacillin, and rifamycin	[46]
***Paenibacillus cucumis***	Novel sp. isolated from a cucumber plant, Gram-positive, rod-shaped bacteria, Optimum temperature for growth is 28–30 °C	[47]
***Rhizobium pusense***	Novel sp. isolated from the rhizosphere of chickpea, Gram-negative, rod-shaped bacteria, Able to grow at 16–41 °C, Main human pathogen within Rhizobium sp.	[48]
***Bacillus thuringiensis***	Gram-positive, rod-shaped bacteria, Natural occurring, soil borne bacteria, Used commercially to control insects important to agriculture and public health, Insecticidal genes from *Bacillus thuringiensis* have been incorporated into several major crops, rendering them insect resistant, Susceptible to streptomycin, chloramphenicol, rifampicin, neomycin, lincomycin, monomycin, kanamycin, and resistant to ampicillin and polymyxin, Resistant to tetracycline	[49]
***Sphingobacterium multivorum***	Gram negative bacteria, Isolated from soil, plants, food and water sources and hospitals, Rarely cause disease in humans, Infection has primarily been reported in immunocompromised patient such as patients with diabetes mellitus, end-stage renal disease patients who are undergoing hemodialysis, patients with malignant cancer who are undergoing chemotherapy, chronic alcoholic liver disease, HIV patients, or cystic fibrosis patients, Bacteremia and acute meningitis also caused by this bacterium, Susceptible to trimethoprim sulfamethoxazole and ciprofloxacin but resistant to aminoglycosides, One reported case showed that the organism was resistant to ampicillin	[50]
***Microbacterium paraoxydans***	Novel sp. isolated from the fish Nile tilapia in Mexico, causes disease in fish, Gram positive bacteria, grow aerobically at 20, 37, and 40°C, Causes bacteremia in immunocompromised patients	[51]
***Bacillus cereus***	Gram positive, toxin-producing bacteria, Commonly found in the environment (soil, vegetation and can be present in foods), Associated with food poisoning, Increasingly reported to be a cause of serious and potentially fatal non gastrointestinal tract infections, Produces tissue destructive exoenzymes, Resistant to penicillin and susceptible to clindamycin, vancomycin, gentamicin, chloramphenicol, and erythromycin	[52]
***Exiguobacterium profundum***	novel sp. moderately thermophilic, lactic acid-producing bacterium isolated from a deep-sea hydrothermal vent, Gram positive bacteria, Grows optimally at 45°C	[53]
***Exiguobacterium indicum***	novel sp.a psychrophilic bacteria isolated from the Hamta glacier of the Himalayan mountain ranges of India, Gram positive, rod-shaped bacteria, Grows at 5–30 °C, Resistant to norfloxacin, cotrimoxazole, clindamycin, doxycycline, sulfamethoxazole, amoxicillin, amikacin, nalidixic acid, nitrofurantoin and colistin, Sensitive to tobramycin, lomefloxacin, roxithromycin, ciprofloxacin, penicillin, cefoperazone, vancomycin, cefuroxime, lincomycin, cefotaxime, cefazolin, kanamycin, novobiocin, chloramphenicol, ampicillin, tetracycline, streptomycin, erythromycin, bacitracin, gentamicin G, polymyxin B, oleandomycin, spectinomycin, rifampicin and carbenicillin.	[54]
***Bacillus aryabhattai***	Novel sp., First isolated and identified from cryotubes used to collect air samples, Dweller of the East Kolkata Wetlands, but its source is not proved, A plant growth promoting rhizobacterium, Gram positive bacteria	[55]
***Bacillus pseudomycoides***	Novel sp., Gram positive bacteria, Optimum growth temperature is 28°C	[56]
***Pseudomonas stutzeri***	Widely distributed in the environment, Human Pathogen, proposed as a model organism for denitrification studies, Participate in the degradation of pollutants or interact with toxic metals, Infection has been reported in association with bacteremia/septicemia, bone infection, eye infection, skin infection, urinary tract infection and ventriculitis	[57]
***Fictibacillus enclensis***	Novel sp. isolated from marine sediment, Gram positive bacteria	[58]

### Heavy metal tolerance test of bacteria isolated from Fijian bivalves and sediments

Heavy metal tolerance tests were conducted for aerobic bacteria and human pathogens isolated from Fijian bivalves’ soft tissues, gills, and river sediments. A total of 20 aerobic bacteria and 27 human pathogens from the soft tissues were evaluated (S1 Table). Among the 20 aerobic bacteria, strains S3ST013, S3ST018, S3ST019, and S3ST021 were tolerant to 1 mM Zn; however, the remaining strains were sensitive at 1mM, and all strains were sensitive at concentrations of 3 mM, 5 mM, and 10 mM. At 1 mM Cu, only strains SIST002, S2ST011, and S3ST021 were sensitive, while the rest were tolerant. All aerobic strains exhibited sensitivity to Cu at 3 mM, 5 mM, and 10 mM. Furthermore, all strains were sensitive to Cd at all tested concentrations. Regarding Cr and Pb, all strains except SIST002 and S2ST011 were tolerant at 1 mM, but all became sensitive at 5 mM and 10 mM. Of the 27 human pathogen strains, *Vibrio* spp. (S3ST041) exhibited tolerance to Zn at all concentrations, while the remaining strains were sensitive at 5 mM and 10 mM. At 1 mM Cu, all strains were tolerant except for S3ST031; however, all strains were sensitive at 5 mM and 10mM. All pathogen strains were sensitive to Cd across all concentrations. At 1 mM Cr and Pb, all strains were tolerant except for S3ST031. While a few strains maintained tolerance at 3 mM and 5 mM Cr, all strains were sensitive to Pb at 3 mM and above (S1 Table).

Out of 33 bacterial strains isolated and purified from the gills, 17 were identified as aerobic bacteria and 16 as human pathogens (S2 Table). Most aerobic isolates showed tolerance to Zn up to 1 mM, and few exhibited up to 3 mM. In contrast, only four aerobic isolates tolerated 1 mM Cu, while strain S1G002 exhibited partial tolerance. All aerobic strains were sensitive to Zn at ≥ 5 mM, to Cu at ≥ 3 mM, and to Cd across all tested concentrations. However, these isolates exhibited elevated tolerance to Cr and Pb, with growth observed at concentrations up to 5 mM (S2 Table). Among the human pathogens, 10 strains tolerated Zn and Cu at 1 mM, with a few maintaining tolerances up to 3 mM. Similar to soft tissues, all pathogen strains were highly susceptible to Cd at all concentrations. Most pathogens tolerated Cr up to 3 mM, and seven strains maintained tolerance up to 5 mM. Furthermore, human pathogens showed strong tolerance to Pb, with the majority of stains surviving at concentrations up to 5 mM. Critically, no bacterial isolates from the gills survived at the maximum concentrations of 10 mM for any tested heavy metal (S2 Table). Overall, soft tissue isolates demonstrated low heavy metal tolerance, primarily restricted to ≤ 1 mM. Similarly, gill-associated bacteria displayed weak tolerance to Zn, Cu, and Cd, but exhibited distinctly higher tolerance profiles for Cr and Pb.

A total of 37 bacterial strains; compromising 16 aerobic bacteria and 21 human pathogens, were isolated from the river sediments for heavy metal tolerance testing (S3 Table). Most aerobic isolates showed tolerance to Zn, Cu, Cr, and Pb at 1 mM; to Cu, Cr, and Pb at 3 mM; and to Cr and Pb at 5 mM. Only a few aerobic strains tolerated 3 mM Zn, and all were universally susceptible to Cd. Among the 21human pathogens, all strains exhibited tolerance to 1 mM Cu, Cr, Pb, whereas *Klebsiella* spp. (S1S023) was uniquely sensitive to 1 mM Zn. The majority of pathogen strains maintained tolerance to Zn, Cu, Cr, and Pb at 3 mM, displayed tolerance to Cr and Pb at 5 mM, and were partially tolerant to 5 mM Cu. Notably, several pathogens strains (Coliforms S1S026, S2S032, S3S039 and *E*. *coli* S3S038) demonstrated extreme tolerance to 10 mM Cr, and the Coliforms S3S039 strain tolerated 10 mM Pb. Additionally, two strains; S1S027 (*Vibrio* spp.) and S2S033 (*Klebsiella* spp.) displayed tolerance to 1 mM Cd, while all remaining pathogen isolates were susceptible to Cd (S3 Table).

### Antibiotic resistance test of bacteria isolated from Fijian bivalves and sediments

Bacterial resistance profiles across various antibiotics concentrations were evaluated (S4-S6 Table). The aerobic bacterial strains *Leclercia adecarboxylata* S1ST003 and *Pseudomonas nitroreducens* S1ST004 exhibited complete resistance to all tested concentrations of rifampicin, vancomycin, and penicillin. Similarly, strain S3ST019 demonstrated resistance across all tested concentrations of rifampicin, penicillin, streptomycin, and tetracycline. Strains S1ST003, S1ST004, S3ST015, and S3ST019 displayed multidrug resistant (MDR) phenotypes at various antibiotic concentrations, even though most aerobic isolates were susceptible to penicillin, streptomycin, and tetracycline.

Among the human pathogens, most strains exhibited resistance to rifampicin, penicillin, and vancomycin at 100 µg/L, as well as to streptomycin and tetracycline at the same concentration. However, variation was observed with streptomycin: three pathogen strains were resistant at 100 µg/l, several were partially resistant at 75 µg/l, and the remaining strains were only resistant at 25 µg/l. For tetracycline, only four strains maintained resistance at 100 µg/l, while the others displayed resistance at lower thresholds (75, 50, and 25 µg/l) or were entirely susceptible. Thermotolerant *E*. *coli* showed robust resistance to rifampicin, penicillin, and streptomycin at all tested concentrations. Overall, most human pathogenic strains displayed multidrug resistant activities at various concentrations across multiple antibiotics.

In summary, the majority of aerobic bacteria isolated from soft tissues remained susceptible to broad-spectrum antibiotic such as rifampicin, vancomycin, streptomycin, and tetracycline, suggesting these as viable treatment options. In contrast, many of human pathogens resisted these broad-spectrum agents; while they were universally resistant to rifampicin and vancomycin, they retained susceptibility to streptomycin and tetracycline. Finally, the narrow-spectrum antibiotic penicillin was ineffective against the tested human pathogens (S4 Table).

Compared to soft tissue isolates, gill-associated bacteria showed greater antibiotic resistance. Most aerobic strains resisted up to 100 μg/L of vancomycin, penicillin, and streptomycin, while displaying resistance thresholds at 25, 50, or 75 µg/l for rifampicin and streptomycin. Strains S1G005, S2G007, S2G008, S3G013, S3G014, S3G016, and S3G019, exhibited multidrug resistant (MDR) phenotypes at various antibiotic concentrations. Similarly, human pathogens isolated from the gills demonstrated widespread resistance to rifampicin, vancomycin, and penicillin at 100 µg/l, whereas their resistance thresholds for streptomycin and tetracycline varied among 25, 50, or 75 µg/l. The majority of these pathogens resisted at least four antibiotics, with Coliform strain S3G033 exhibiting complete resistance to all tested concentrations of all antibiotics. Overall, the human pathogen strains showed more robust multidrug resistant profiles against various antibiotics than the aerobic bacterial strains (S5 Table).

Aerobic bacteria isolated river sediments demonstrated lower overall resistance to broad-spectrum antibiotics such as rifampicin, vancomycin, streptomycin, and tetracycline. Furthermore, only a minority of these strains exhibited resistance to the narrow-spectrum antibiotic penicillin. A notable exception was S3S018 strain, which displayed complete resistance to vancomycin, penicillin, streptomycin, and tetracycline across all tested concentrations. Along with S3S018, strains S1S001 and S2S016 displayed distinct multidrug resistance (MDR) phenotypes at various antibiotic concentrations. In contrast, human pathogens from the sediments exhibited higher resistance profiles for rifampicin, vancomycin, and penicillin. While the majority of these pathogenic strains remained susceptible to streptomycin and tetracycline, a small subset displayed resistance to both agents. Overall, most human pathogen strains demonstrated robust multidrug resistance activities across different antibiotic classes (S6 Table). Conclusively, sediment-derived aerobic bacteria were predominantly susceptible to the tested antibiotics, whereas the human pathogens showed significantly greater resistance.

Numerous bacterial strains isolated from soft tissues, gills, and sediments exhibited multidrug resistance (MDR) phenotypes across the tested matrices (Table 5). Specifically, seven strains from soft tissues, comprising two aerobic isolates and five human pathogens, demonstrated pan-resistance to all five antibiotics (rifampicin, vancomycin, penicillin, streptomycin, and tetracycline). Similarly, seven gill-associated strains exhibited resistance to all five agents, which included three aerobic strains and four human pathogens. Within the sediment samples, eight strains demonstrated complete resistance across all five antibiotic classes, consisting of two aerobic isolates and six human pathogens.

**Table 5:** Antibiotic resistant bacterial strains from fijian bivalves and sediments. Highlighted strains are resistant to all five antibiotics. S1 – Site 1, S2 – Site 2, S3 – Site 3.

Antibiotics used in this study	Antibiotic’s mode of action on bacteria	Resistant strains isolated from this study		
		Soft Tissues	Gills	Sediments
**Rifampicin**	Inhibition of RNA synthesis	S1ST003, **S1ST004**, S3ST015, **S3ST018**, S3ST019, S1ST022, S1ST023, S1ST024, S1ST025, S2ST026, **S2ST027**, S2ST028, S2ST029, S3ST030, S3ST032, S3ST033, S3ST034, S3ST036, S3ST039, **S3ST041**, **S1ST046**, **S2ST047**, **S3ST048**	S1G003,S1G005, S2G007,**S2G008**, S2G009,**S3G013**. **S3G014**,S3G018, S3G019,**S1G022**, S1G024,S2G026, S2G027,S2G028, **S3G029**,**S3G030**, S3G031,S3G032, **S3G033**,S1G034, S2G035, S3G036	**S2S016**,**S3S018**, **S1S022**,S1S023, S1S024,S1S026, **S1S027**,S2S029, S2S031,**S2S032**, S2S033,**S3S036**, S3S037,**S3S038**, **S3S039**,S1S042, S2S043, S3S044
**Vancomycin**	Inhibition of cell wall synthesis	S1ST003, **S1ST004**, S3ST015, **S3ST018**, S3ST021, S1ST022, S1ST023, S1ST024, S1ST025, S2ST026, **S2ST027**, S2ST028, S2ST029, S3ST030, S3ST032, S3ST033, S3ST034, S3ST036, S3ST037, S3ST039, **S3ST041**, **S1ST046**, **S2ST047**, **S3ST048**	S1G001,S1G002, S1G005,S2G007, **S2G008**,S2G009, S3G012,**S3G013**, **S3G014**,S3G016, S3G018,S3G019, S3G020,**S1G022**, S1G024,S1G025, S2G026,S2G027, S2G028,**S3G029**, **S3G030**,S3G031, S3G032,**S3G033**, S1G034,S2G035, S3G036	S1S005,S1S006, **S2S016**,**S3S018**, S3S019,**S1S022**, S1S023,S1S024, S1S026,**S1S027**, S1S028,S2S029, S2S031,**S2S032**, S2S033,S2S034, S3S035,**S3S036**, S3S037,**S3S038**, **S3S039**,S1S042, S2S043, S3S044
**Penicillin**	Inhibition of cell wall synthesis	S1ST003**,S1ST004**, S2ST006,**S3ST018**, S3ST019,S1ST022, S1ST023,S1ST024, S1ST025,S2ST026, **S2ST027**,S2ST028, S2ST029,S3ST030, S3ST031,S3ST032, S3ST033,S3ST034, S3ST035,S3ST036, S3ST038,S3ST039, S3ST040,**S3ST041**, **S1ST046**,**S2ST047**, **S3ST048**	S1G005,S2G007, **S2G008**,S2G009, S3G012,**S3G013**, **S3G014**,S3G016, S3G020,**S1G022**, S1G024,S2G026, S2G027,S2G028, **S3G029**,**S3G030**, S3G031,S3G032, **S3G033**,S1G034, S2G035, S3G036	S1S001,S1S002, S1S008,S1S009, S1S010,S1S012, **S2S016**,**S3S018**, S3S020,**S1S022**, S1S023,S1S024, S1S026,**S1S027**, S1S028,S2S029, S2S031,**S2S032**, S2S033,S2S034, S3S035,**S3S036**, S3S037,**S3S038**, **S3S039**,S3S040, S1S042,S2S043, S3S044
**Streptomycin**	Inhibition of protein synthesis	S1ST004,**S2ST008**, S3ST015,S3ST017, **S3ST018**,S3ST019, S2ST026,**S2ST02**7, S3ST031,S3ST034, S3ST035,S3ST036, S3ST037,S3ST038, S3ST039,S3ST040, **S3ST041**,S3ST042, S3ST043,S3ST044, S3ST045,**S1ST046**, **S2ST047**, **S3ST048**	S1G002,S1G003, S1G004,**S2G008**, S2G009,S3G012, **S3G013**,**S3G014**, S3G016,S3G019, S3G020,**S1G022**, S1G024,S1G025, S2G026,**S3G029**, **S3G030**,S3G032, **S3G033**	S1S003,S2S013, S23015,**S2S016**, **S3S018**,**S1S022**, **S1S027**,**S2S032**, **S3S036**,S3S037, **S3S038**,**S3S039,** S3S040
**Tetracyclin**	Inhibition of protein synthesis	S1ST003,**S1ST004**, S2ST009,S2ST010, **S3ST018**,S3ST019, S3ST021,S1ST022, S1ST023,**S2ST027**, S2ST029,S3ST030, **S3ST04**1,**S1ST046**, **S2ST047**, **S3ST048**	**S2G008**,S3G012, **S3G013**,**S3G014**, S3G016,S3G018, S3G019,**S1G022**, S2G027,S2G028, **S3G029**,**S3G030**, S3G031, **S3G033**	S1S001,S1S007, S1S010,S1S012, **S2S016**,**S3S018**, **S1S022**,**S1S027**, S2S031,**S2S032**, S2S033,**S3S036**, **S3S038**, **S3S039**

## Discussion

In the Pacific Islands, coastal and freshwater resources support the sustenance and socioeconomic livelihoods of numerous local communities [59,60]. However, anthropogenic disturbances and ecosystem alterations in aquatic environments have increased over the years, potentially elevating environmental contamination. Current understanding of contamination levels and food safety regarding freshwater resources remains limited, particularly concerning pathogenic contaminants, pathogen diversity, and antibiotic and heavy metal resistance. Local communities along the Ba River in Fiji continue to consume and utilize resources from the river for the daily needs [61,62] without prior knowledge of the associated potential health risks. In this study, analysis of Ba River sediments and the bivalve *B. violacea* revealed that the highest total aerobic bacterial count occurred in sediments, followed by soft tissues and gills. Site 1 was the most heavily polluted by microorganisms. Elevated aerobic bacterial loads in waters and bivalves typically indicate contamination from anthropogenic developments. For instance, Bainivalu [63] noted elevated levels of total aerobic bacteria in water samples collected at the Fiji Sugar Corporation (FSC) sugar mill outfall along Qawa River, Labasa, Fiji, identifying the sugar mill effluent as the source of the high heterotrophic load. This aligns with our findings, where the highest aerobic bacterial count was recorded at Site 1, located behind the Rarawai Sugar Mill. Sugar mill effluent plays a key role in increasing bacterial populations in the aquatic environments as it is rich in organic matter, serving as an ideal nutrient source for bacteria. Additionally, Hatha [64] and Singh [65] attributed prominent aerobic bacterial loads in bivalves from the Suva market, Fiji, to contaminated source waters. Our results, however, showed a much higher aerobic bacterial load in sediments than in the bivalves.

Faecal coliforms are well-documented contaminants in seafood and aquatic environments, and bivalves are known to bioaccumulate these bacteria. Although our study revealed low levels of faecal coliforms in both bivalves and sediments, this finding diverges from previous studies that typically reported higher concentrations [7,66–69]. This discrepancy may be attributed to regular dredging activities within the Ba River [70]. Dredging operations can remove the surface layer of sediments, which typically harbours significantly higher concentrations of faecal coliforms than deeper layers [69]. The removal of this contaminated surface layer may explain the lower faecal coliform levels observed in our study. Conversely, Hatha [64] reported coliform levels in bivalves from the Suva market that were comparable to our findings. Coliform contamination in bivalves has been widely documented across Fiji, Korea, Brazil and Philippines [65,70–72]. While most strains of *E*. *coli* are harmless, certain strains cause serious foodborne illnesses, primarily transmitted to humans through the ingestion of contaminated foods, such as raw or undercooked ground meat products, raw milk, and raw vegetables and sprouts [73]. Our results showed that *E*. *coli* was present at low concentrations in both bivalve and sediment samples. Notably, however, thermotolerant *E*. *coli* was detected in soft tissues and sediments across all sampling sites, likely due to an influx of untreated sewage into the river.

Interestingly, human pathogens were also present in the bivalves and sediments. *Vibrio* spp. is a bacterial group responsible for most human diseases associated with aquatic environments and seafood consumption, with *V*. *cholerae*, *V*. *parahaemolyticus*, *V*. *vulnificus* and *V*. *alginolyticus* being the most common pathogenic species [74]. These bacteria are transmitted to humans through the ingestion of contaminated water or raw and undercooked seafood [75]. Our findings indicated that bivalves from Site 1 and sediments from all three sites contained *Vibrio* spp. Similarly, previous studies have reported the presence of *Vibrio* spp. in bivalves and river sediments [64,76,77] demonstrating that *Vibrio* spp. are persistent in aquatic environments and can accumulate through the food chain.

Faecal *Streptococcus*, which resides in the intestines of humans and warm-blooded animals [78], was detected in bivalves from all three locations. Site 1 showed a significantly higher concentration, strongly suggesting targeted faecal contamination. Previous works have similarly documented faecal streptococci in bivalves from the River Blackwater Estuary, UK [79] Ashtamudi Lake, India [7], and Izmir Bay, Turkey [80]. Furthermore, major foodborne pathogens such as *Salmonella* spp. and *Shigella* spp. were detected in bivalves and sediments across all three sites. *Salmonella* spp. and *Shigella* spp. impact millions of people annually, leading to acute gastrointestinal illness [81] characterized by headaches, fever, cough, nausea, vomiting, constipation, abdominal pain, chills, rose spots, bloody and mucoid stools, and diarrhoea [1]. Jeamsripong [82] reported their occurrence in oysters from the Phang Nga Estuary, Thailand, while Collin [83] identified them in clams collected from Maputo Bay, Mozambique. Additionally, Patel and Payne [84] detected these pathogens in sediments from St. John’s Harbour, indicating their potential for ingestion by bivalves residing in contaminated environments.

This study also revealed that *Klebsiella* spp. exhibited a comparable abundance across all three sampling sites, with bivalves demonstrating significantly higher bacterial loads than the surrounding sediments. *Klebsiella* spp. are well-documented opportunistic pathogens capable of causing severe human infections, including pneumonia, bacteraemia, wound/surgical site infections, meningitis, and urinary tract infections [85]. The elevated *Klebsiella* spp. loads in these bivalves suggest localised faecal contamination of the coastal environment. Previous research has confirmed the presence of *K*. *pneumoniae* in bivalve mollusks, highlighting its widespread distribution in aquatic environments [85–87]. These findings underscore the potential public health risks associated with consuming contaminated seafood.

Bacteria identification *via* 16s rRNA gene sequencing revealed a diverse microbial community, including *Rhodococcus hoagie*, *Curtobacterium citreum*, *Leclercia adecarboxylata*, *Pseudomonas nitroreducens*, *Pseudomonas stutzeri*, and several Bacillus species (*B. toyonensis*, *B. thuringiensis*, *B. cereus*, *B. aryabhattai*, and *B. pseudomycoides*). Other identified species included *Microbacterium laevaniformans*, *M. natoriense*, *M. paraoxydans*, *Acinetobacter kookii*, *Paenibacillus barcinonensis*, *P. cucumis*, *Staphylococcus edaphicus*, *Streptomyces katrae*, *Rhizobium pusense*, *Sphingobacterium multivorum*, *Exiguobacterium profundum*, *Exiguobacterium indicum*, and *Fictibacillus enclensis*. that the spatial distribution of these was noteworthy: *Rhodococcus*, *Curtobacterium*, and *Paenibacillus* were unique to soft tissues, while *Staphylococcus*, *Streptomyces*, *Rhizobium*, and *Sphingobacterium* were found exclusively in the gills. *Exigubacterium* and *Fictibacillus* were restricted to sediments. Conversely, *Leclercia*, *Pseudomonas*, *Microbacteria*, and *Acinetobacter* were present in both soft tissues and gills, whereas *Bacillus* was ubiquitous across all samples. Interestingly, existing literature has not yet confirmed the presence of several of these specific bacteria in bivalves. Of the 23 identified species, six are known human pathogens, eight are multidrug-resistant (MRD), nine are novel species, and four have applications in agriculture, livestock, or aquaculture. These finding mirror research by Rout [88], who identified *Pseudomonadota*, *Actinomycetota*, *Bacteroidota*, and *Bacillota* as the dominant phyla in the Ganges River. The presence of these pathogens in the Ba River suggests significant anthropogenic contamination, likely stemming from human and animal faecal matter, household effluents, and agriculture or industrial runoff. Consequently, local populations consuming resources from the Ba River face unquantified health risks.

The sequencing results further reinforce the evidence of anthropogenic impact, particularly through the prevalence of MDR bacteria. In this study, 15 strains; seven from tissues/gills and eight from sediments; exhibited resistance to all five tested antibiotics. Notably, several human pathogens, including Shigella spp., *Vibrio* spp., thermotolerant *E. coli*, *Salmonella* spp., faecal *Streptococcus*, and *Enterococcus* spp., displayed MDR properties. These findings align with previous studies on mussels and mollusks where resistance to streptomycin, vancomycin, and tetracycline was documented [89,90]. The presence of MDR bacteria in bivalves and sediments is a serious public concern, as it facilitates the spread of antibiotic-resistance genes (ARGs) within the aquatic ecosystem as Rout [91] and Rout [92] reported, the distribution of ARGs is non-uniform and influenced by a complex interplay of environmental conditions and local antibiotic usage.

Natural detoxification is essential for regulating heavy metal concentrations in aquatic environments. Heavy metal-tolerant bacteria can persist in contaminated sites and may play a vital role in bioremediation [93–96]. This study that while most isolates tolerated Cu and Pb up to 1 mM and Cr up to 3 mM, tolerance to Zn was generally limited. A notable exception was strain S3ST41 (*Vibrio* spp.), which tolerated all tested Zn concentrations. While most isolates were susceptible to Cd, strains S1S027 (*Vibrio* spp.) and S2S033 (*Klebsiella* spp.), exhibited tolerance to 1 mM. Interestingly, gill-associated bacteria displayed higher tolerance to Cr and Pb, while human pathogens showed a broader range of resistance, surviving elevated concentrations of multiple metals.

To mitigate health risks, consumers should ensure bivalves are through cooked and avoid raw consumption. Implementing depuration systems, such as the closed circulatory sand biofilter demonstrated by Singh [65] and Singh [97], can effectively reduce bacterial loads to safe levels. Given the projected rise in antibiotic resistance, exploring alternative treatments is critical. Fijian ethnomedicine and traditional herbal remedies represent a promising frontier for novel antimicrobial chemical entities [98,99]. Furthermore, future studies should investigate seasonal variations to better understand how environmental changes influence contamination patterns.

## Conclusion

This study highlights that Site 1, located behind the Rarawai sugar mill, is heavily contaminated. The microbial load was highest in soft tissues, followed by sediments, and gills. The presence of pathogens such as *Vibrio* spp., faecal *Streptococcus*, and thermotolerant *E. coli* is particularly concerning.. Furthermore, human pathogens exhibited significantly higher tolerance to both heavy metals and antibiotics than aerobic bacteria. While their metal tolerance suggests potential bioremediation, their MDR status poses a severe challenge for clinical treatment. Therefore, stringent depuration and high temperature cooking are essential to ensure the safety of bivalves harvested from these waters.

## Supporting information

S1 Table Heavy Metal Tolerance of Bacteria from Soft Tissues. (DOCX)

S2 Table Heavy Metal Tolerance of Bacteria from Gills. (DOCX)

S3 Table Heavy Metal Tolerance of Bacteria from Sediments. (DOCX)

S4 Table Antibiotic Resistance Test of Bacteria from Soft Tissues. (DOCX)

S5 Table Antibiotic Resistant Test of Bacteria from Gills. (DOCX)

S6 Table Antibiotic Resistant Test of Bacteria from Sediments. (DOCX)
