# Supplementary material for: Antibiotic-resistant pathogens and heavy metal-tolerant bacteria in Batissa violacea: A study from the Ba River, Fiji
Source: PLoS One. 2026 Sep 25;21(9):e0359203. doi: 10.1371/journal.pone.0359203 (PMC13614535; doi:10.1371/journal.pone.0359203)
Supplement: Supplementary file 2 [file pone.0359203.s002.docx]

**S2 Table. Heavy Metal Tolerance of Bacteria from Gills.**

|  | **Heavy Metals (mM)** | | | | | | | | | | | | | | | | | | | | |
| --- | --- | --- | --- | --- | --- | --- | --- | --- | --- | --- | --- | --- | --- | --- | --- | --- | --- | --- | --- | --- | --- |
| **Strain** |  |  | **Zn** |  |  |  | **Cu** |  |  |  | **Cd** |  |  |  | **Cr** |  | **Pb** | | | |  |
|  | **1** | **3** | **5** | **10** | **1** | **3** | **5** | **10** | **1** | **3** | **5** | **10** | **1** | **3** | **5** | **10** | **1** | **3** | **5** | **10** |  |
| **S1G001** | **+** | - | - | - | - | - | - | - | - | - | - | - | **+** | **+** | **+** | - | **+** | **+** | **+** | - |  |
| **S1G002** | **+** | - | - | - | **+*** | - | - | - | - | - | - | - | **+** | **+** | - | - | **+** | **+** | **+** | - |  |
| **S1G003** | **+** | - | - | - | - | - | - | - | - | - | - | - | **+** | **+** | - | - | **+** | **+** | - | - |  |
| **S1G004** | **+** | - | - | - | - | - | - | - | - | - | - | - | **+** | **+** | **+** | - | **+** | **+** | - | - |  |
| **S1G005** | **+** | - | - | - | **+** | - | - | - | - | - | - | - | **+** | **+** | **+** | - | **+** | **+** | **+** | - |  |
| **S2G007** | **+** | **+** | - | - | **+** | - | - | - | - | - | - | - | **+** | **+** | **+** | - | **+** | **+** | - | - |  |
| **S2G008** | - | - | - | - | - | - | - | - | - | - | - | - | - | - | - | - | - | - | - | - |  |
| **S2G009** | **+** | - | - | - | - | - | - | - | - | - | - | - | **+** | **+** | - | - | **+** | **+** | **+** | - |  |
| **S2G010** | - | - | - | - | - | - | - | - | - | - | - | - | - | - | - | - | - | - | - | - |  |
| **S3G012** | **+** | **+** | - | - | - | - | - | - | - | - | - | - | **+** | **+** | - | - | **+** | **+** | - | - |  |
| **S3G013** | **+** | - | - | - | - | - | - | - | - | - | - | - | **+** | **+** | **+** | - | **+** | **+** | **+** | - |  |
| **S3G014** | **+** | **+** | - | - | - | - | - | - | - | - | - | - | **+** | **+** | **+*** | - | **+** | **+** | - | - |  |
| **S3G016** | **+** | **+** | - | - | - | - | - | - | - | - | - | - | **+** | **+** | **+*** | - | **+** | **+** | - | - |  |
| **S3G017** | - | - | - | - | - | - | - | - | - | - | - | - | - | - | - | - | - | - | - | - |  |
| **S3G018** | **+** | **+** | - | - | **+** | - | - | - | - | - | - | - | **+** | **+** | - | - | **+** | **+** | **+** | - |  |
| **S3G019** | **+** | **+** | - | - | **+** | - | - | - | - | - | - | - | **+** | **+** | - | - | **+** | **+** | **+** | - |  |
| **S3G020** | **+** | **+** | - | - | - | - | - | - | - | - | - | - | **+** | **+** | **+** | - | **+** | **+** | **+** | - |  |
| **S1G021** | - | - | - | - | - | - | - | - | - | - | - | - | **+** | **+** | - | - | - | - | - | - |  |
| **S1G022** | **+** | - | - | - | **+** | **+** | - | - | - | - | - | - | **+** | **+** | **+** | - | **+** | **+** | **+** | - |  |
| **S1G023** | - | - | - | - | - | - | - | - | - | - | - | - | - | - | - | - | - | - | - | - |  |
| **S1G024** | **+** | - | - | - | **+** | - | - | - | - | - | - | - | **+** | **+** | **+** | - | **+** | **+** | **+** | - |  |
| **S1G025** | **+** | - | - | - | **+** | - | - | - | - | - | - | - | **+** | **+** | - | - | **+** | **+** | **+** | - |  |
| **S2G026** | **+** | - | - | - | **+** | - | - | - | - | - | - | - | **+** | **+** | - | - | **+** | **+** | **+** | - |  |
| **S2G027** | **+** | - | - | - | **+** | - | - | - | - | - | - | - | **+** | **+** | **+** | - | **+** | **+** | **+** | - |  |
| **S2G028** | - | - | - | - | - |  | - | - | - | - | - | - | - | - | - | - | - | - | - | - |  |
| **S3G029** | **+** | **+** | - | - | **+** | **+** | - | - | - | - | - | - | **+** | **+** | **+** | - | **+** | **+** | **+** | - |  |
| **S3G030** | **+** | **+** | - | - | **+** | - | - | - | - | - | - | - | **+** | **+** | - | - | **+** | **+** | - | - |  |
| **S3G031** | **+** | **+** | - | - | **+** | - | - | - | - | - | - | - | **+** | **+** | - | - | **+** | **+** | - | - |  |
| **S3G032** | **+** | **+** | - | - | **+** | **+** | - | - | - | - | - | - | **+** | **+** | **+** | - | **+** | **+** | **+** | - |  |
| **S3G033** | **+** | **+** | - | - | **+** | **+** | - | - | - | - | - | - | **+** | **+** | **+** | - | **+** | **+** | **+** | - |  |
| **S1G034** | - | - | - | - | - | - | - | - | - | - | - | - | **+** | **+** | - | - | **+** | **+** | **+** | - |  |
| **S2G035** | - | - | - | - | - | - | - | - | - | - | - | - | **+** | **+** | - | - | **+** | **+** | **+** | - |  |
| **S3G036** | - | - | - | - | - | - | - | - | - | - | - | - | **+** | **+** | - | - | **+** | **+** | **+** | - |  |

***+** Tolerant; - Sensitive; **+*** Partially tolerant
