# Supplementary material for: Antibiotic-resistant pathogens and heavy metal-tolerant bacteria in Batissa violacea: A study from the Ba River, Fiji
Source: PLoS One. 2026 Sep 25;21(9):e0359203. doi: 10.1371/journal.pone.0359203 (PMC13614535; doi:10.1371/journal.pone.0359203)
Supplement: Supplementary file 3 [file pone.0359203.s003.docx]

**S3 Table. Heavy Metal Tolerance of Bacteria from Sediments.**

|  | **Heavy Metals (mM)** | | | | | | | | | | | | | | | | | | | |
| --- | --- | --- | --- | --- | --- | --- | --- | --- | --- | --- | --- | --- | --- | --- | --- | --- | --- | --- | --- | --- |
| **Strain** |  |  | **Zn** |  |  |  | **Cu** |  |  |  | **Cd** |  |  |  | **Cr** |  | **Pb** | | | |
|  | **1** | **3** | **5** | **10** | **1** | **3** | **5** | **10** | **1** | **3** | **5** | **10** | **1** | **3** | **5** | **10** | **1** | **3** | **5** | **10** |
| **S1S001** | **+** | **+** | - | - | **+** | **+** | - | - | - | - | - | - | **+** | **+** | **+** | - | **+** | **+** | **+** | - |
| **S1S002** | **+** | - | - | - | **+** | - | - | - | - | - | - | - | **+** | **+** | **+** | - | **+** | **+** | **+** | - |
| **S1S003** | **+** | - | - | - | **+** | **+*** | - | - | - | - | - | - | **+** | **+** | **+** | - | **+** | **+** | - | - |
| **S1S005** | **+** | - | - | - | **+** | **+** | - | - | - | - | - | - | **+** | **+** | **+** | - | **+** | **+** | - | - |
| **S1S006** | **+** | **+** | - | - | **+** | **+** | - | - | - | - | - | - | **+** | **+** | **+** | - | **+** | **+** | - | - |
| **S1S007** | - | - | - | - | **+** | - | - | - | - | - | - | - | **+** | **+** | **+** | - | **+** | **+** | **+** | - |
| **S1S008** | **+** | **+** | - | - | **+** | **+** | - | - | - | - | - | - | **+** | **+** | **+** | - | **+** | **+** | **+** | - |
| **S1S009** | - | - | - | - | - | - | - | - | - | - | - | - | **+** | **+** | - | - | **+** | **+** | - | - |
| **S1S010** | - | - | - | - | **+** | - | - | - | - | - | - | - | **+** | **+** | **+** | - | **+** | **+** | **+** | - |
| **S1S012** | - | - | - | - | **+** | **+** | - | - | - | - | - | - | **+** | **+** | **+** | - | **+** | **+** | **+** | - |
| **S2S013** | **+** | **+** | **+** | - | **+** | **+** | - | - | - | - | - | - | **+** | **+** | **+** | - | **+** | **+** | **+** | - |
| **S23015** | **+** | **+** | **+** | - | **+** | - | - | - | - | - | - | - | **+** | **+** | **+** | - | **+** | **+** | **+** | - |
| **S2S016** | **+** | - | - | - | **+** | **+** | - | - | - | - | - | - | **+** | **+** | **+*** | - | **+** | **+** | - | - |
| **S3S018** | **+** | **+** | **+** | - | **+** | **+** | - | - | - | - | - | - | **+** | **+** | **+** | - | **+** | **+** | - | - |
| **S3S019** | **+** | **+** | - | - | **+** | **+** | - | - | - | - | - | - | **+** | **+** | **+** | - | **+** | **+** | **+** | - |
| **S3S020** | - | - | - | - | **+** | - | - | - | - | - | - | - | **+** | **+** | **+** | - | **+** | **+** | **+** | - |
| **S1S022** | **+** | - | - | - | **+** | **+** | - | - | - | - | - | - | **+** | **+** | **+** | - | **+** | **+** | **+** | - |
| **S1S023** | - | - | - | - | **+** | - | - | - | - | - | - | - | **+** | **+** | **+** | - | **+** | **+** | - | - |
| **S1S024** | **+** | - | - | - | **+** | - | - | - | - | - | - | - | **+** | **+** | **+** | - | **+** | **+** | - | - |
| **S1S026** | **+** | **+** | - | - | **+** | **+** | **+*** | - | - | - | - | - | **+** | **+** | **+** | **+*** | **+** | **+** | **+** | - |
| **S1S027** | **+** | **+** | - | - | **+** | **+** | - | - | **+** | - | - | - | **+** | **+** | **+** | - | **+** | **+** | **+** | - |
| **S1S028** | **+** | - | - | - | **+** | **+** | - | - | - | - | - | - | **+** | **+** | **+** | - | **+** | **+** | - | - |
| **S2S029** | **+** | - | - | - | **+** | - | - | - | - | - | - | - | **+** | **+** | **+** | - | **+** | **+** | - | - |
| **S2S031** | **+** | **+** | - | - | **+** | **+** | **+*** | - | - | - | - | - | **+** | **+** | **+** | - | **+** | **+** | **+** | - |
| **S2S032** | **+** | **+** | - | - | **+** | **+** | **+*** | - | - | - | - | - | **+** | **+** | **+** | **+** | **+** | **+** | **+** | - |
| **S2S033** | **+** | **+** | - | - | **+** | **+** | - | - | **+** | - | - | - | **+** | **+** | **+** | - | **+** | **+** | **+** | - |
| **S2S034** | **+** | - | - | - | **+** | **+** | - | - | - | - | - | - | **+** | **+** | **+** | - | **+** | **+** | - | - |
| **S3S035** | **+** | - | - | - | **+** | **+** | - | - | - | - | - | - | **+** | **+** | **+** | - | **+** | **+** | - | - |
| **S3S036** | **+** | - | - | - | **+** | **+** | - | - | - | - | - | - | **+** | **+** | **+** | - | **+** | **+** | **+** | - |
| **S3S037** | **+** | **+** | - | - | **+** | - | - | - | - | - | - | - | **+** | **+** | **+** | - | **+** | **+** | **+** | - |
| **S3S038** | **+** | **+** | - | - | **+** | **+** | - | - | - | - | - | - | **+** | **+** | **+** | **+*** | **+** | **+** | **+** | - |
| **S3S039** | **+** | **+** | - | - | **+** | **+** | - | - | - | - | - | - | **+** | **+** | **+** | **+** | **+** | **+** | **+** | **+*** |
| **S3S040** | **+** | - | - | - | **+** | **+** | - | - | - | - | - | - | **+** | **+** | **+** | - | **+** | **+** | - | - |
| **S3S041** | **+** | **+** | **+*** |  | **+** | **+*** | - | - | - | - | - | - | **+** | **+** | **+** | - | **+** | **+** | **+** | - |
| **S1S042** | **+** | - | - | - | **+** | **+*** | - | - | - | - | - | - | **+** | **+** | **+*** | - | **+** | **+** | **+** | - |
| **S2S043** | **+** | - | - | - | **+** | **+*** | - | - | - | - | - | - | **+** | **+** | **+*** | - | **+** | **+** | **+** | - |
| **S3S044** | **+** | - | - | - | **+** | **+*** | - | - | - | - | - | - | **+** | **+** | **+*** | - | **+** | **+** | **+** | - |

***+** Tolerant; - Sensitive; **+*** Partially tolerant.
