# Supplementary material for: Antibiotic-resistant pathogens and heavy metal-tolerant bacteria in Batissa violacea: A study from the Ba River, Fiji
Source: PLoS One. 2026 Sep 25;21(9):e0359203. doi: 10.1371/journal.pone.0359203 (PMC13614535; doi:10.1371/journal.pone.0359203)
Supplement: Supplementary file 4 [file pone.0359203.s004.docx]

**S4 Table. Antibiotic Resistance Test of Bacteria from Soft Tissues.**

|  | **Antibiotic (µg/l)** | | | | | | | | | | | | | | | | | | | | | |
| --- | --- | --- | --- | --- | --- | --- | --- | --- | --- | --- | --- | --- | --- | --- | --- | --- | --- | --- | --- | --- | --- | --- |
| **Strain** | **Rifampicin** | | | | **Vancomycin** | | | | **Penicillin** | | | | **Streptomycin** | | | | | **Tetracycline** | | | | |
|  | **25** | **50** | **75** | **100** | **25** | **50** | **75** | **100** | **25** | **50** | **75** | **100** | **25** | **50** | **75** | **100** | **25** | | **50** | **75** | **100** |  |
| **S1ST001** | - | - | - | - | - | - | - | - | - | - | - | - | - | - | - | - | **+** | | **+** | - | - |  |
| **S1ST002** | - | - | - | - | - | - | - | - | - | - | - | - | - | - | - | - | - | | - | - | - |  |
| **S1ST003** | **+** | **+** | **+** | **+** | **+** | **+** | **+** | **+** | **+** | **+** | **+** | **+** | - | - | - | - | **+*** | | - | - | - |  |
| **S1ST004** | **+** | **+** | **+** | **+** | **+** | **+** | **+** | **+** | **+** | **+** | **+** | **+** | **+** | **+** | **+*** | - | **+** | | **+** | **+** | **+** |  |
| **S2ST005** | **+** | - | - | - | - | - | - | - | - | - | - | - | **+** | - | - | - | - | | - | - | - |  |
| **S2ST006** | - | - | - | - | - | - | - | - | **+** | **+** | **+** | **+** | **+** | **+** | **+*** | - | **+** | | - | - | - |  |
| **S2ST007** | - | - | - | - | - | - | - | - | - | - | - | - | **+** | - | - | - | **+** | | **+** | - | - |  |
| **S2ST008** | - | - | - | - | - | - | - | - | - | - | - | - | **+** | **+** | **+** | **+** | - | | - | - | - |  |
| **S2ST009** | - | - | - | - | - | - | - | - | **+** | - | - | - | - | - | - | - | **+** | | **+** | **+** | - |  |
| **S2ST010** | - | - | - | - | - | - | - | - | **+** | - | - | - | - | - | - | - | **+** | | **+** | **+** | - |  |
| **S2ST011** | - | - | - | - | - | - | - | - | - | - | - | - | - | - | - | - | - | | - | - | - |  |
| **S3ST012** | - | - | - | - | - | - | - | - | - | - | - | - | **+** | - | - | - | - | | - | - | - |  |
| **S3ST013** | - | - | - | - | - | - | - | - | - | - | - | - | **+** | - | - | - | - | | - | - | - |  |
| **S3ST014** | - | - | - | - | - | - | - | - | - | - | - | - | - | - | - | - | - | | - | - | - |  |
| **S3ST015** | **+** | **+** | **+** | **+** | **+** | **+** | **+** | **+** | - | - | - | - | **+** | **+** | **+** | **+** | **+** | | - | - | - |  |
| **S3ST016** | - | - | - | - | - | - | - | - | - | - | - | - | - | - | - | - | - | | - | - | - |  |
| **S3ST017** | - | - | - | - | - | - | - | - | - | - | - | - | **+** | **+** | **+** | **+** | - | | - | - | - |  |
| **S3ST018** | **+** | **+** | - | - | **+** | **+** | **+** | **+** | **+** | **+** | **+** | **+** | **+** | **+** | **+*** | - | **+** | | **+** | **+** |  |  |
| **S3ST019** | **+** | **+** | **+** | **+** | - | - | - | - | **+** | **+** | **+** | **+** | **+** | **+** | **+** | **+** | **+** | | **+** | **+** | **+** |  |
| **S3ST021** | - | - | - | - | **+** | **+** | **+** | - | **+*** | - | - | - | **+*** | - | - | - | **+** | | **+** | **+** | **+*** |  |
| **S1ST022** | **+** | **+** | **+** | **+** | **+** | **+** | **+** | **+** | **+** | **+** | **+** | **+** | **+** | - | - | - | **+** | | **+** | **+** | - |  |
| **S1ST023** | **+** | **+** | **+** | **+** | **+** | **+** | **+** | **+** | **+** | **+** | **+** | **+** | **+*** | - | - | - | **+** | | **+** | **+** | - |  |
| **S1ST024** | **+** | **+** | **+** | **+** | **+** | **+** | **+** | **+** | **+** | **+** | **+** | **+** | - | - | - | - | - | | - | - | - |  |
| **S1ST025** | **+** | **+** | **+** | **+** | **+** | **+** | **+** | **+** | **+** | **+** | **+** | **+** | **+** | - | - | - | **+** | | - | - | - |  |
| **S2ST026** | **+** | **+** | **+** | **+** | **+** | **+** | **+** | **+** | **+** | **+** | **+** | **+** | **+** | **+*** | - | - | **+** | | - | - | - |  |
| **S2ST027** | **+** | **+** | **+** | **+** | **+** | **+** | **+** | **+** | **+** | **+** | **+** | **+** | **+** | **+** | - | - | **+** | | **+** | **+** | **+** |  |
| **S2ST028** | **+** | **+** | **+** | **+** | **+** | **+** | **+** | **+** | **+** | **+** | **+** | **+** | **+** | - | - | - | **+** | | - | - | - |  |
| **S2ST029** | **+** | **+** | **+** | **+** | **+** | **+** | **+** | **+** | **+** | **+** | **+** | **+** | **+** | - | - | - | **+** | | **+** | **+** | **+*** |  |
| **S3ST030** | **+** | **+** | **+** | **+** | **+** | **+** | **+** | **+** | **+** | **+** | **+** | **+** | **+** | - | - | - | **+** | | **+** | **+** | **+*** |  |
| **S3ST031** | - | - | - | - | **+** | - | - | - | **+** | **+** | **+** | - | **+** | **+** | **+*** | - | - | | - | - | - |  |
| **S3ST032** | **+** | **+** | **+** | **+** | **+** | **+** | **+** | **+** | **+** | **+** | **+** | **+** | **+** | - | - | - | **+** | | - | - | - |  |
| **S3ST033** | **+** | **+** | **+** | **+** | **+** | **+** | **+** | **+** | **+** | **+** | **+** | **+** | - | - | - | - | - | | - | - | - |  |
| **S3ST034** | **+** | **+** | **+** | **+** | **+** | **+** | **+** | **+** | **+** | **+** | **+** | **+** | **+** | **+*** | - | - | **+** | | - | - | - |  |
| **S3ST035** | - | - | - | - | **+** | **+** | - | - | - | - | - | - | **+** | **+*** | - | - | - | | - | - | - |  |
| **S3ST036** | **+** | **+** | - | - | **+** | **+** | **+** | **+** | **+** | **+** | **+** | **+** | **+** | **+*** | - | - | **+** | | - | - | - |  |
| **S3ST037** | **+** | - | - | - | **+** | **+** | **+** | - | **+*** | - | - | - | **+** | **+** | **+** | **+** | **+*** | | - | - | - |  |
| **S3ST038** | - | - | - | - | - | - | - | - | **+** | **+** | **+** | **+** | **+** | **+*** | - | - | - | | - | - | - |  |
| **S3ST039** | **+** | **+** | - | - | **+** | **+** | **+** | **+** | **+** | **+** | **+** | **+** | **+** | **+** | **+** | **+** | **+*** | | - | - | - |  |
| **S3ST040** | - | - | - | - | - | - | - | - | **+** | **+** | **+** | **+** | **+** | **+** | - | - | - | | - | - | - |  |
| **S3ST041** | **+** | **+** | **+** | **+** | **+** | **+** | **+** | **+** | **+** | **+** | **+** | **+** | **+** | **+** | **+** | **+** | **+** | | **+** | **+** | - |  |
| **S3ST042** | - | - | - | - | - | - | - | - | - | - | - | - | **+** | **+*** | - | - | - | | - | - | - |  |
| **S3ST043** | - | - | - | - | - | - | - | - | - | - | - | - | **+** | **+*** | - | - | - | | - | - | - |  |
| **S3ST044** | - | - | - | - | - | - | - | - | - | - | - | - | **+** | **+*** | - | - | - | | - | - | - |  |
| **S3ST045** | - | - | - | - | - | - | - | - | - | - | - | - | **+** | **+** | **+*** | - | - | | - | - | - |  |
| **S1ST046** | **+** | **+** | **+** | **+** | **+** | **+** | **+** | **+** | **+** | **+** | **+** | **+** | **+** | **+*** | - | - | **+** | | **+** | - | - |  |
| **S2ST047** | **+** | **+** | **+** | **+** | **+** | **+** | **+** | **+** | **+** | **+** | **+** | **+** | **+** | **+*** | - | - | **+** | | **+** | - | - |  |
| **S3ST048** | **+** | **+** | **+** | **+** | **+** | **+** | **+** | **+** | **+** | **+** | **+** | **+** | **+** | **+*** | - | - | **+** | | **+** | - | - |  |

***+** Resistant; - Sensitive; **+*** Partially resistant
