# Supplementary material for: Antibiotic-resistant pathogens and heavy metal-tolerant bacteria in Batissa violacea: A study from the Ba River, Fiji
Source: PLoS One. 2026 Sep 25;21(9):e0359203. doi: 10.1371/journal.pone.0359203 (PMC13614535; doi:10.1371/journal.pone.0359203)
Supplement: Supplementary file 5 [file pone.0359203.s005.docx]

**S5 Table. Antibiotic Resistant Test of Bacteria from Gills.**

|  | **Antibiotic (µg/l)** | | | | | | | | | | | | | | | | | | | | |
| --- | --- | --- | --- | --- | --- | --- | --- | --- | --- | --- | --- | --- | --- | --- | --- | --- | --- | --- | --- | --- | --- |
| **Strain** | **Rifampicin** | | | | **Vancomycin** | | | | **Penicillin** | | | | **Streptomycin** | | | | **Tetracycline** | | | | |
|  | **25** | **50** | **75** | **100** | **25** | **50** | **75** | **100** | **25** | **50** | **75** | **100** | **25** | **50** | **75** | **100** | **25** | **50** | **75** | **100** |  |
| **S1G001** | - | - | - | - | **+** | **+** | - | - | - | - | - | - | **+** | **+** | - | - | - | - | - | - |  |
| **S1G002** | - | - | - | - | **+** | **+** | **+** | - | - | - | - | - | **+** | **+*** | - | - | - | - | - | - |  |
| **S1G003** | **+** | **+** | **+** | - | **+** | - | - | - | - | - | - | - | **+** | **+** | **+** | **+** | - | - | - | - |  |
| **S1G004** | - | - | - | - | - | - | - | - | - | - | - | - | **+** | **+** | **+** | **+** | - | - | - | - |  |
| **S1G005** | **+** | **+** | **+** | - | **+** | **+** | **+** | **+** | **+** | **+** | **+** | **+** | - | - | - | - | **+** | - | - | - |  |
| **S2G007** | **+** | **+** | **+** | - | **+** | **+** | **+** | **+** | **+** | **+** | **+** | **+** | **+** | - | - | - | **+** | - | - | - |  |
| **S2G008** | **+** | **+** | - | - | **+** | **+** | **+** | **+** | **+** | **+** | **+** | **+** | **+** | **+** | - | - | **+** | **+** | - | - |  |
| **S2G009** | **+** | **+** | **+** | - | **+** | **+** | **+** | **+** | **+** | **+** | **+** | **+** | **+** | **+** | **+** | **+** | - | - | - | - |  |
| **S2G010** | - | - | - | - | - | - | - | - | - | - | - | - | - | - | - | - | - | - | - | - |  |
| **S3G012** | - | - | - | - | **+** | **+** | **+** | **+** | **+** | **+** | **+** | **+** | **+** | **+** | **+** | **+** | **+** | **+** | **+** | - |  |
| **S3G013** | **+** | **+** | **+** | - | **+** | **+** | **+** | **+** | **+** | **+** | **+** | **+** | **+** | **+** | - | - | **+** | **+** | **+** | - |  |
| **S3G014** | **+** | **+** | - | - | **+** | **+** | **+** | **+** | **+** | **+** | **+** | **+** | **+** | **+** | **+** | **+** | **+** | **+** | **+** | - |  |
| **S3G016** | - | - | - | - | **+** | **+** | **+** | **+** | **+** | **+** | **+** | **+** | **+** | **+** | **+** | **+** | **+** | **+** | **+** | - |  |
| **S3G017** | - | - | - | - | - | - | - | - | - | - | - | - | - | - | - | - | - | - | - | - |  |
| **S3G018** | **+** | **+** | **+** | - | **+** | **+** | **+** | **+** | - | - | - | - | **+** | - | - | - | **+** | **+** | **+** | **+** |  |
| **S3G019** | **+** | **+** | **+** | - | **+** | **+** | **+** | **+** | - | - | - | - | **+** | **+** | - | - | **+** | **+** | **+** | **+** |  |
| **S3G020** | **+** | - | - | - | **+** | **+** | **+** | **+** | **+** | **+** | **+** | **+** | **+** | **+** | **+** | **+** | - | - | - | - |  |
| **S1G021** | **+** | - | - | - | - | - | - | - | - | - | - | - | - | - | - | - | - | - | - | - |  |
| **S1G022** | **+** | **+** | **+** | **+** | **+** | **+** | **+** | **+** | **+** | **+** | **+** | **+** | **+** | **+** | **+*** | - | **+** | **+** | **+** | **+** |  |
| **S1G023** | - | - | - | - | - | - | - | - | - | - | - | - | - | - | - | - | - | - | - | - |  |
| **S1G024** | **+** | **+** | **+** | **+** | **+** | **+** | **+** | **+** | **+** | **+** | **+** | **+** | **+** | **+** | **+** | **+** | **+** | - | - | - |  |
| **S1G025** | **+** | - | - | - | **+** | **+** | **+** | **+** | **+** | - | - | - | **+** | **+** | **+*** | - | - | - | - | - |  |
| **S2G026** | **+** | **+** | **+** | **+** | **+** | **+** | **+** | **+** | **+** | **+** | **+** | **+** | **+** | **+** | **+** | **+** | - | - | - | - |  |
| **S2G027** | **+** | **+** | **+** | **+** | **+** | **+** | **+** | **+** | **+** | **+** | **+** | **+** | - | - | - | - | **+** | **+** | - | - |  |
| **S2G028** | **+** | **+** | **+** | **+** | **+** | **+** | **+** | **+** | **+** | **+** | **+** | **+** | - | - | - | - | **+** | **+** | **+** | **+** |  |
| **S3G029** | **+** | **+** | **+** | **+** | **+** | **+** | **+** | **+** | **+** | **+** | **+** | **+** | **+** | **+** | **+** | - | **+** | **+** | **+** | **+** |  |
| **S3G030** | **+** | **+** | **+** | **+** | **+** | **+** | **+** | **+** | **+** | **+** | **+** | **+** | **+** | **+** | - | - | **+** | **+** | **+** | **+** |  |
| **S3G031** | **+** | **+** | **+** | **+** | **+** | **+** | **+** | **+** | **+** | **+** | **+** | **+** | **+** | - | - | - | **+** | **+** | **+** | **+** |  |
| **S3G032** | **+** | **+** | **+** | **+** | **+** | **+** | **+** | **+** | **+** | **+** | **+** | **+** | **+** | **+** | **+*** | - | **+** | - | - | - |  |
| **S3G033** | **+** | **+** | **+** | **+** | **+** | **+** | **+** | **+** | **+** | **+** | **+** | **+** | **+** | **+** | **+** | **+** | **+** | **+** | **+** | **+** |  |
| **S1G034** | **+** | **+** | **+** | **+** | **+** | **+** | **+** | - | **+** | **+** | **+** | - | - | - | - | - | - | - | - | - |  |
| **S2G035** | **+** | **+** | **+** | **+** | **+** | **+** | **+** | - | **+** | **+** | **+** | - | - | - | - | - | - | - | - | - |  |
| **S3G036** | **+** | **+** | **+** | **+** | **+** | **+** | **+** | - | **+** | **+** | **+** | - | - | - | - | - | - | - | - | - |  |

***+** Resistant; - Sensitive; **+*** Partially resistant
