# Supplementary material for: Antibiotic-resistant pathogens and heavy metal-tolerant bacteria in Batissa violacea: A study from the Ba River, Fiji
Source: PLoS One. 2026 Sep 25;21(9):e0359203. doi: 10.1371/journal.pone.0359203 (PMC13614535; doi:10.1371/journal.pone.0359203)
Supplement: Supplementary file 6 [file pone.0359203.s006.docx]

**S6 Table. Antibiotic Resistant Test of Bacteria from Sediments.**

|  | **Antibiotic (µg/l)** | | | | | | | | | | | | | | | | | | | | |
| --- | --- | --- | --- | --- | --- | --- | --- | --- | --- | --- | --- | --- | --- | --- | --- | --- | --- | --- | --- | --- | --- |
| **Strain** | **Rifampicin** | | | | **Vancomycin** | | | | **Penicillin** | | | | **Streptomycin** | | | | **Tetracycline** | | | | |
|  | **25** | **50** | **75** | **100** | **25** | **50** | **75** | **100** | **25** | **50** | **75** | **100** | **25** | **50** | **75** | **100** | **25** | **50** | **75** | **100** |  |
| **S1S001** | - | - | - | - | **+** | - | - | - | **+** | **+** | **+** | **+** | **+** | - | - | - | **+** | **+** | **+** | **+** |  |
| **S1S002** | - | - | - | - | - | - | - | - | **+** | **+** | **+** | **+** | - | - | - | - | - | - | - | - |  |
| **S1S003** | - | - | - | - | - | - | - | - | - | - | - | - | **+** | **+** | - | - | - | - | - | - |  |
| **S1S005** | - | - | - | - | **+** | **+** | - | - | - | - | - | - | **+** | - | - | - | - | - | - | - |  |
| **S1S006** | - | - | - | - | **+** | **+** | **+** | - | - | - | - | - | - | - | - | - | - | - | - | - |  |
| **S1S007** | - | - | - | - | - | - | - | - | **+** | - | - | - | **+** | - | - | - | **+** | **+** | **+** | **+** |  |
| **S1S008** | - | - | - | - | **+** | - | - | - | **+** | **+** | **+** | **+** | **+** | - | - | - | **+** | - | - | - |  |
| **S1S009** | - | - | - | - | **+*** | - | - | - | **+** | **+** | **+** | **+** | - | - | - | - | - | - | - | - |  |
| **S1S010** | - | - | - | - | - | - | - | - | **+** | **+*** | - | - | **+*** | - | - | - | **+** | **+** | **+** | **+** |  |
| **S1S012** | - | - | - | - | - | - | - | - | **+** | **+*** | - | - | - | - | - | - | **+** | **+** | **+** | **+** |  |
| **S2S013** | - | - | - | - | **+** | - | - | - | - | - | - | - | **+** | **+** | - | - | - | - | - | - |  |
| **S23015** | - | - | - | - | **+** | - | - | - | - | - | - | - | **+** | **+** | - | - | - | - | - | - |  |
| **S2S016** | **+** | **+** | **+** | - | **+** | **+** | **+** | **+** | **+** | **+** | **+** | **+** | **+** | **+** | - | - | **+** | - | - | - |  |
| **S3S018** | **+** | **+** | - | - | **+** | **+** | **+** | **+** | **+** | **+** | **+** | **+** | **+** | **+** | **+** | **+** | **+** | **+** | **+** | **+** |  |
| **S3S019** | - | - | - | - | **+** | **+** | - | - | - | - | - | - | **+** | - | - | - | - | - | - | - |  |
| **S3S020** | - | - | - | - | - | - | - | - | **+** | **+*** | - | - | - | - | - | - | **+** | **+** | **+** | **+** |  |
| **S1S022** | **+** | **+** | **+** | **+** | **+** | **+** | **+** | **+** | **+** | **+** | **+** | **+** | **+** | **+** | - | - | **+** | **+** | - | - |  |
| **S1S023** | **+** | **+** | - | - | **+** | **+** | **+** | **+** | **+** | **+** | **+** | **+** | - | - | - | - | **+** | - | - | - |  |
| **S1S024** | **+** | **+** | **+** | **+** | **+** | **+** | **+** | **+** | **+** | **+** | **+** | **+** | - | - | - | - | **+** | - | - | - |  |
| **S1S026** | **+** | **+** | **+** | **+** | **+** | **+** | **+** | **+** | **+** | **+** | **+** | **+** | - | - | - | - | **+** | - | - | - |  |
| **S1S027** | **+** | **+** | **+** | **+** | **+** | **+** | **+** | **+** | **+** | **+** | **+** | **+** | **+** | **+** | **+** | **+** | **+** | **+** | - | - |  |
| **S1S028** | - | - | - | - | **+** | **+** | **+** | **+** | **+** | **+** | **+** | **+** | **+** | - | - | - | - | - | - | - |  |
| **S2S029** | **+** | **+** | **+** | - | **+** | **+** | **+** | **+** | **+** | **+** | **+** | - | - | - | - | - | **+** | - | - | - |  |
| **S2S031** | **+** | **+** | **+** | **+** | **+** | **+** | **+** | **+** | **+** | **+** | **+** | **+** | - | - | - | - | **+** | **+** | - | - |  |
| **S2S032** | **+** | **+** | **+** | **+** | **+** | **+** | **+** | **+** | **+** | **+** | **+** | **+** | **+** | **+** | **+** | **+** | **+** | **+** | **+** | - |  |
| **S2S033** | **+** | **+** | **+** | **+** | **+** | **+** | **+** | **+** | **+** | **+** | **+** | **+** | **+** | **+** | - | - | **+** | **+** | **+** | **+** |  |
| **S2S034** | - | - | - | - | **+** | **+** | - | - | **+** | **+** | **+** | **+** | **+** | - | - | - | - | - | - | - |  |
| **S3S035** | - | - | - | - | **+** | **+** | **+** | **+** | **+** | **+** | **+** | **+** | **+** | - | - | - | - | - | - | - |  |
| **S3S036** | **+** | **+** | **+** | **+** | **+** | **+** | **+** | **+** | **+** | **+** | **+** | **+** | **+** | **+** | - | - | **+** | **+** | **+** |  |  |
| **S3S037** | **+** | **+** | **+** | **+** | **+** | **+** | **+** | **+** | **+** | **+** | **+** | **+** | **+** | **+** | - | - | **+** | - | - | - |  |
| **S3S038** | **+** | **+** | **+** | **+** | **+** | **+** | **+** | **+** | **+** | **+** | **+** | **+** | **+** | **+** | - | - | **+** | **+** | **+** | **+** |  |
| **S3S039** | **+** | **+** | **+** | **+** | **+** | **+** | **+** | **+** | **+** | **+** | **+** | **+** | **+** | **+** | - | - | **+** | **+** | - | - |  |
| **S3S040** | - | - | - | - | **+** | - | - | - | **+** | **+** | **+** | **+** | **+** | **+** | - | - | - | - | - | - |  |
| **S3S041** | - | - | - | - | - | - | - | - | **+** | - | - | - | - | - | - | - | - | - | - | - |  |
| **S1S042** | **+** | **+** | **+** | **+** | **+** | **+** | **+** | **+** | **+** | **+** | **+** | **+** | - | - | - | - | - | - | - | - |  |
| **S2S043** | **+** | **+** | **+** | **+** | **+** | **+** | **+** | **+** | **+** | **+** | **+** | **+** | - | - | - | - | - | - | - | - |  |
| **S3S044** | **+** | **+** | **+** | **+** | **+** | **+** | **+** | **+** | **+** | **+** | **+** | **+** | - | - | - | - | - | - | - | - |  |

***+** Resistant; - Sensitive; **+*** Partially resistant
