# Supplementary material for: Antibiotic-resistant pathogens and heavy metal-tolerant bacteria in Batissa violacea: A study from the Ba River, Fiji
Source: PLoS One. 2026 Sep 25;21(9):e0359203. doi: 10.1371/journal.pone.0359203 (PMC13614535; doi:10.1371/journal.pone.0359203)
Supplement: Supplementary file 7 [file pone.0359203.s007.docx]

PONE-D-26-07821
Antibiotic-Resistant Pathogens and Heavy Metal-Tolerant Bacteria in Batissa violacea: A Study from the Ba River, Fiji

22^nd^ August, 2026

Dear Editor,

We have prepared a response to reviewer comments for your consideration. The responses for each comment are included in the table below.

| **Journal Requirements** | | |
| --- | --- | --- |
| 1. Please ensure that your manuscript meets PLOS ONE's style requirements, including those for file naming. The PLOS ONE style templates can be found at | The manuscript was formatted as per the requirements of the journal. | |
| 2. In your Methods section, please provide additional information regarding the permits you obtained for the work. Please ensure you have included the full name of the authority that approved the field site access and, if no permits were required, a brief statement explaining why. | Line 112-114 A brief statement was included. | |
| 3. Please state what role the funders took in the study. | The funders had no role in study design, data collection and analysis, decision to publish, or preparation of the manuscript. | |
| 4. We note that Figure 1 in your submission contain [map/satellite] images which may be copyrighted. | Figure one was amended to remove any possible copyrighted material. The included maps were created using the ‘R’ software. | |
| 5. If the reviewer comments include a recommendation to cite specific previously published works, please review and evaluate these publications to determine whether they are relevant and should be cited. There is no requirement to cite these works unless the editor has indicated otherwise. | Some citations were recommended. Only relevant citations were included. | |
| **Review Comments to the Author** | | |
| **Reviewer #1:** The research addresses an important public health issue linking antibiotic resistance and heavy metal tolerance in aquatic environments. The manuscript is well-structured and provides comprehensive experimental approaches (MPN, culture, antibiotic testing, 16S rRNA). However, the manuscript requires clarification, deeper analysis, and improved scientific clarity before publication. The authors should consider the following points before finalizing the manuscript. | | |
| 1. The study is relevant, but the novelty is not clearly mentioned. Authors should highlight how this work differs from previous studies in similar aquatic systems. | | Thank you very much for your suggestions. The authors have included at the end of introduction section of the manuscript in red. |
| 2. The sample size justification and replication strategy are insufficient. Include a sampling map with clearer labeling of sampling sites. The limitations of the study (e.g., culturable bacteria only) should be mentioned in the discussion section of the article. | | Thank you very much for your suggestions. The authors have revised in the methodology and discussion sections of the manuscript in red. |
| 3. The antibiotic susceptibility method (agar dilution) should be justified against standard methods (e.g., CLSI guidelines). | | The authors have made the revisions in the methodology, which are shown in red. Thank you. |
| 4. Results are mostly descriptive; quantitative comparisons and statistical interpretation should be required. | | Thank you very much for your suggestions. The authors have revised the results section of the manuscript in red color. |
| 5. Figures and tables need clear labeling, units, and improved readability. | | Figures and labels were revised for better clarity. |
| 6. The authors should develop phylogenetic trees and a deeper discussion of microbial diversity is necessary. | | Discussion section was revised for better clarity and readability. Genetic sequencing of the samples was done through an arrangement and the sequences required for phylogenetic tree development are no longer accessible or available to the authors. |
| 7. The claim of multidrug resistance (MDR) needs a clearer definition (e.g., ≥3 antibiotic classes). | | The authors have made the revisions, which are shown in red. Thank you. |
| 8. Results should be compared with regional/global AMR trends. | | Thank you very much for your suggestions. The authors have made the revisions, which are shown in red. |
| 9. The ecological relevance of tested concentrations (1–10 mM) should be justified. | | Further justification was added to the Methods in red.  Due to Site 1’s proximity to a known effluent discharge from the sugar mill, it is likely to experience elevated levels of pollutants, including heavy metals. In this regard, the selected concentration range of heavy metals (1–10 mM) was used to assess bacterial tolerance and adaptive responses to metal stress. |
| 10. Discuss possible co-selection mechanisms between ARGs and MRGs more critically. | | Thank you very much for your suggestions. The authors have revised the manuscript in red. |
| 11. Several typographical errors need correction. | | The authors have revised the manuscript in red. Thank you. |
| 12. Standardize terms (e.g., Vibrio spp., E. coli, formatting inconsistencies). | | The authors have revised the manuscript in red. Thank you. |
| 13. Improve figure captions for better understanding. | | Figure captions were revised for better clarity. |
| **Reviewer #2**: The manuscript addresses an important topic; however, it requires substantial revision in terms of experimental clarity, statistical rigor, and interpretation of results. The current version is largely descriptive and lacks strong mechanistic insights. | | |
| 1. Insufficient information on experimental protocols, sequencing depth, and data processing. | | More details were added in methods and results section. |
| 2. Statistical methods are not well described. | | More details on statistical methods were added in red. |
| 3. Lack of clarity on assumptions, replicates, and validation of results. | | More information on statistical procedures were added to the methods in red. |
| 4. Conclusions are overstated relative to the data. | | Revised in red color. Thank you. |
| 5. Weak correlations are interpreted as strong relationships. | | Changes were made in the results and discussions in red to reflect the results more fairly. |
| 6. Kindly insert the latest references and remove old references. | | More recent references were incorporated. |
| 7. Kindly describe more in the discussion and introduction. | | Revised in red color. Thank you very much. |
| 8. Cite a few important papers in the discussion and introduction, PMID: 40789383, 36508095, 38663298, 32683080, 38136769, 33178147, 5750913 | | Thank you very much for your suggestions. The authors have cited relevant articles from the suggestions in the Discussion and Introduction sections. |
| **Minor Concerns** | | |
| Improve figure quality and legends. | | Figures and captions were revised for better clarity. |
| Correct grammatical and typographical errors. | | Revised in red color. Thank you. |
| In the manuscript, some gaps/unnecessary spaces are present. Kindly correct them. | | These were corrected. |
